# The determinants of expert opinion in the development of care pathways: insights from an exploratory cluster analysis

**DOI:** 10.1186/s12913-023-09139-7

**Published:** 2023-03-03

**Authors:** Matteo Ratti, Osvaldo Milicia, Riccardo Rescinito, Ellen Coeckelberghs, Deborah Seys, Kris Vanhaecht, Massimiliano Panella

**Affiliations:** 1 Department of Translational Medicine (DiMeT), University of Piemonte Orientale (UNIUPO), Via Solaroli,17, 28100 Novara, Italy; 2 Leuven Institute for Healthcare Policy, KU Louvain—University of Leuven, Leuven, Belgium; 3 European Pathway Association, Louvain, Belgium

**Keywords:** Care pathways, Expert opinion, Cluster analysis, Myasthenia gravis, Multiple correspondence analysis, Factorial analysis

## Abstract

**Background:**

We performed a secondary exploratory cluster analysis on the data collected from the validation phase of the study leading to the development of the model care pathway (CP) for Myasthenia Gravis (MG), in which a panel of 85 international experts were asked some characteristics about themselves and their opinion about the model CP. Our aim was to identify which characteristics of the experts play a role in the genesis of their opinion.

**Methods:**

We extracted the questions probing an opinion and those describing a characteristic of the expert from the original questionnaire. We performed a multiple correspondence analysis (MCA) and a subsequent hierarchical clustering on principal component (HCPC) on the opinion variables, integrating the characteristic variables as supplementary (predicted).

**Results:**

After reducing the dimensionality of the questionnaire to three dimensions we noticed that the not-appropriateness judgement of the clinical activities may overlap with the completeness one. From the HCPC it seems that the working setting of the expert may play a crucial role in determining the opinion about the setting of the sub-processes of MG: shifting from a cluster where the experts do not work in sub-specialist settings to one where the experts are working in them, the opinion changes accordingly from a mono-disciplinary setting to a multi-disciplinary one. Another interesting result is that the experience in neuromuscular diseases (NMD) measured in years and the expert typology (whether general neurologist or NMD expert) seem not to contribute significantly to the opinions.

**Conclusions:**

These findings might indicate a poor ability of the expert to discriminate what is not appropriate from what is not complete. Also, the opinion of the expert might be influenced by the working setting, but not by the experience in NMD (as measured in years).

**Supplementary Information:**

The online version contains supplementary material available at 10.1186/s12913-023-09139-7.

## Background

A Care Pathway (CP) is an instrument to connect what is currently known to what is currently delivered to the patient: in brief, it is a way to translate evidence into practice with the aim of minimizing health care costs while providing the best patient-centered care. This methodology has already been applied successfully to many diseases such as hip fractures [1, 2], non-ST elevation myocardial infarction [3], and chronic obstructive pulmonary diseases [4]. One of the first steps towards the development of a CP is to methodically collect the most up-to-date evidences about a disease or a condition [5]. Then, the researcher must extract the clinical activities and then cluster them into a set of Key Interventions (KI), which represents one of the active components of the CP. The local adherence of the health care facility to the KIs (measured with specific process indicators) will ultimately give information about the performance of the health care system and highlight possible evidence-practice gaps. This process is relatively easy for high volume and high-cost conditions, for which there is plenty of literature derived from high-quality level of evidence studies. However, when dealing with rare diseases the evidence is scarce or derived from low-quality sources.In such situations, the opinion of the experts carries a high weight to the whole evidence collected. Most of the recent research about CPs has been focused on the main active ingredients, such as the feedback on the current care process [6], the strategy to improve the current care process, or teamwork [7, 8]. To our knowledge, this is the first attempt to analyze the expert opinion in such a context. We believe that, since the implementation of a CP must consider the local reality, a deeper understanding of the determinants of the expert opinion could be of help for a successful implementation. For these reasons, we decided to perform a secondary exploratory analysis of the data generated by the Delphi survey filled by the experts in the context of our previous study concerning the development of the CP of MG. Our objective was to identify which characteristics of the experts may contribute to the generation of their opinions.

## Materials and methods

### Context

In 2019 the European Pathway Association (EPA) with UCB Pharma conducted a study for developing a Model Care Pathway for Myasthenia Gravis. The study consisted in the following phases: literature review and assessment, process analysis and care pathway design, care pathway validation and care pathway implementation tools. The literature review and assessment process provided 60 evidence based clinical activities, which were then grouped into seven sub-processes: diagnostic (1); pharmacologic management (2); speech, swallow, and dental needs (3); occupational, physical, and respiratory management process (4); psychological needs (5); lifestyle management (6); myasthenic crisis assessment and management (7). The activities were also compiled into 7 sub-process flow charts. In the care pathway validation step, 85 international experts of Myasthenia Gravis were recruited and asked to fill a questionnaire after looking at the sub-process flow charts. In brief, they were asked some general information and, for every subprocess: if it was complete, if it included not appropriate activities, if they spot a bottleneck, and the setting where the sub-process is currently executed. The results showed a high level of agreement among the experts both for completeness and for appropriateness of the proposed CP, and some statistically significant regional differences in the prevalent setting of specific sub-processes: most notably, US experts showed a tendency towards the use of ambulatory setting in case of diagnosis and pharmacological management, whereas for the same sub-process the EU experts preferred the hospital setting. The final step led to the definition of 14 Key Interventions and 24 related process indicators. The full description and results of the study are reported in the relative article [9].

### Secondary analysis

This study is a secondary exploratory cluster analysis of the data collected from the survey filled by the MG experts in the care pathway validation phase. The survey was conducted among 85 experts:43 from United States (US), 35 from the European Union (EU) and 7 from Japan (JP); the whole original questionnaire is reported in Supplementary materials 1. We used the SoGoSurvey platform [10] both to send the surveys and to collect the responses.

### Extraction and recoding of the original answers

From the whole original questionnaire, we extracted the questions regarding general information about the expert (Q1-Q7) and, for each MG CP sub-process: presence of not appropriate activities (Qx8), judgment on completeness (Qx1), presence of bottlenecks (Qx3), how many setting identified where the sub-process is currently executed (Qx6). We labeled the variables as “opinion” if the relative question probed an opinion or “characteristics” if it was related to a characteristic of the expert. For the common settings where the sub-process is currently executed (where the experts were allowed to enter multiple answers), we recoded the answers as “mono-disciplinary” if the expert reported only a single setting for the sub-process (thus giving a judgement of relative certainty), and “multi-disciplinary” otherwise. We excluded the experts who did not fill completely any of the questions extracted, for they did not express an opinion.

### Factorial analysis

We performed a multiple correspondence analysis (MCA) as a dimensionality reduction technique only on the “opinion” variables, integrating the “characteristics” as supplementary (predicted) variables. MCA is a principal component analysis method that transforms categorical data into coordinates of several space dimensions. The main idea shared among all the principal component methods is to describe a dataset by using a small number of uncorrelated variables (dimensions) limiting the loss of information at the least possible value. In brief, these dimensions explain a certain percentage of the total variance. We used the indicator matrix approach, and applied no ventilation level for the variables. After examining the resulting scree plot, the smallest number of dimensions that relates to the largest explained variance were kept for subsequent analysis.

### Hierarchical clustering on principal components (HCPC)

We conducted a hierarchical clustering on the identified principal components (dimensions) using Ward’s agglomeration criterion. This technique decomposes the total variance (labeled as inertia) in between and within group variance such that the growth of the within inertia is minimum at each step. The within inertia therefore characterizes the homogeneity of a single cluster. The clustering hierarchy is then graphically represented by a dendrogram which is indexed by the gain of within-inertia. The algorithm also automatically provides an optimal number of clusters, since we have no prior information or theory about this topic.

### Data analysis

All analysis were conducted with R ver. 4.1.0, R Studio ver.2021.09.2, FactoMineR ver.2.4, FactoExtra ver. 1.0.7 and tidyverse ver. 1.3.1 packages [11–15]. The resultant clusters were described by principal components and by categories. The FactoMineR partition algorithm performs a Cramer v-test (based on Chi-Square) on each variable to detect statistically significant differences among clusters. We considered statistically significant a *p*-value less than 0.05. Since it is an exploratory analysis of a small sample, we also considered quasi-significant a *p*-value between 0.05 and 0.1. In the interpretation of the results, we generated hypothesis only on the basis of the significant and quasi-significant findings.

## Results

We extracted a total of 35 out of 77 original questions for this secondary analysis: 28 probed the expert opinions about the care pathway and 7 concerned the characteristics of the experts. The recoded map of the “Characteristic” questions is reported in Table S4, while the “Opinion” questions are reported in Table S5 in supplementary materials. From the original 85 experts, we excluded 15 because they did not properly fill all the questions, ending with 30 EU experts (42.9%), 4 JP experts (5.7%), and 36 US experts (51.4%), for a total of 70 experts.

### Factorial analysis

**Fig. 1:**
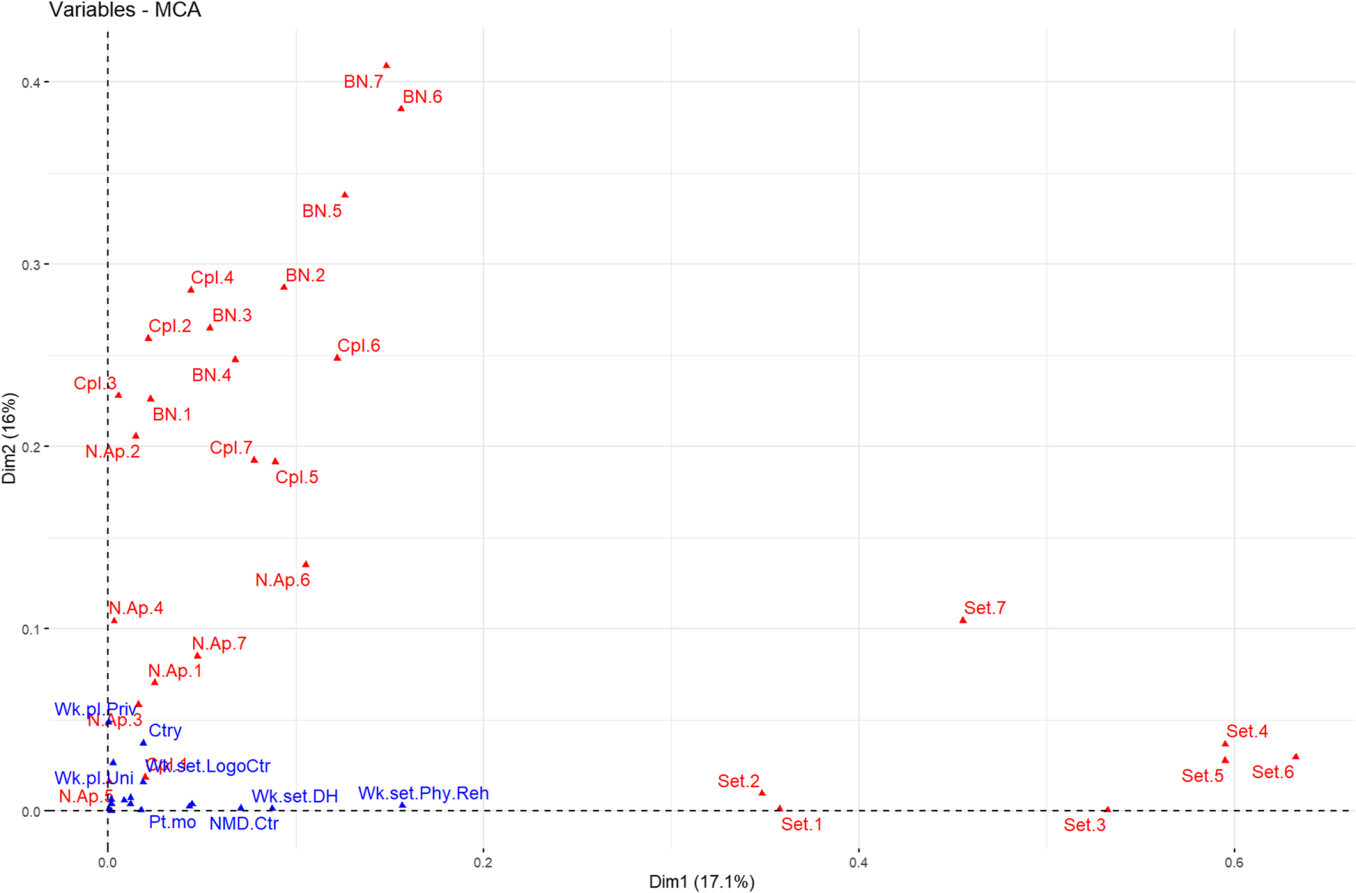
Plot of the squared correlation between the variables and the MCA principal dimensions. The "opinion" variables are in red, the "characteristic" (predicted) variables in blue

The first 10 dimensions extracted by the MCA accounted for 17,1%, 16%, 10.9%, 8.1%, 5.5%, 5.3%, 4.5%, 4.1%, 3.9% and 3% of the total explained variance, respectively. After the scree plot examination, we kept the first three dimensions for further analysis, explaining for 44% of the total variance: Dim1, Dim2 and Dim3. The scree plot of the dimensions is reported in Supplementary materials 2 (Fig. S1). Figure 1 shows the correlation of the variables with the MCA first two principal dimensions.. The first 10 contributors to the three dimensions are reported in Table 1. The contribution of every combination of variable/category to the three dimensions and the relative squared cosine values are reported in Supplementary materials 2 (Table S1). A positive value of dimension 1 seems to detect an opinion of multidisciplinary current setting for many sub-processes. The second dimension appears to describe a predominant judgement of absence of bottlenecks along with a judgment of not completeness of the sub-processes. Finally, Dim3 most likely probes an opinion of not completeness of many sub-processes (predominantly the sub-specialist ones). The N.Ap variables are not significantly represented in the resulting dimensions: therefore, their information is redundant with other variables.

**Table 1:** Top 10 contributors to Dim1, Dim2 and Dim3

	**Dim1** **(var %)**	Dim1** Variable**	**Dim2** **(var %)**	Dim2** Variable**	**Dim3** **(var %)**	Dim3** Variable**
**1**	9.4602	Set.6_multi	6.2757	BN.7_No	11.6302	Cpl.6_No
**2**	9.0767	Set.5_multi	5.9407	Cpl.4_No	10.0131	Cpl.7_No
**3**	8.5980	Set.3_multi	5.4720	Cpl.2_No	9.4347	Cpl.5_No
**4**	8.0098	Set.4_multi	5.4194	BN.6_No	7.8866	Cpl.4_No
**5**	6.2071	Set.1_multi	5.3254	Cpl.6_No	6.6222	Cpl.2_No
**6**	5.8557	Set.7_multi	5.0513	BN.2_No	5.7270	N.Ap.6_Yes
**7**	5.8318	Set.2_multi	4.6630	Cpl.3_No	4.9483	N.Ap.7_Yes
**8**	4.4499	Set.4_mono	4.3389	BN.1_No	4.8405	N.Ap.3_Yes
**9**	3.7841	Set.6_mono	4.2378	BN.3_No	3.6551	Cpl.3_No
**10**	3.6769	Set.7_mono	4.2137	BN.5_No	3.2584	N.Ap.5_Yes

### Hierarchical clustering on principal components (HCPC)

**Fig. 2:**
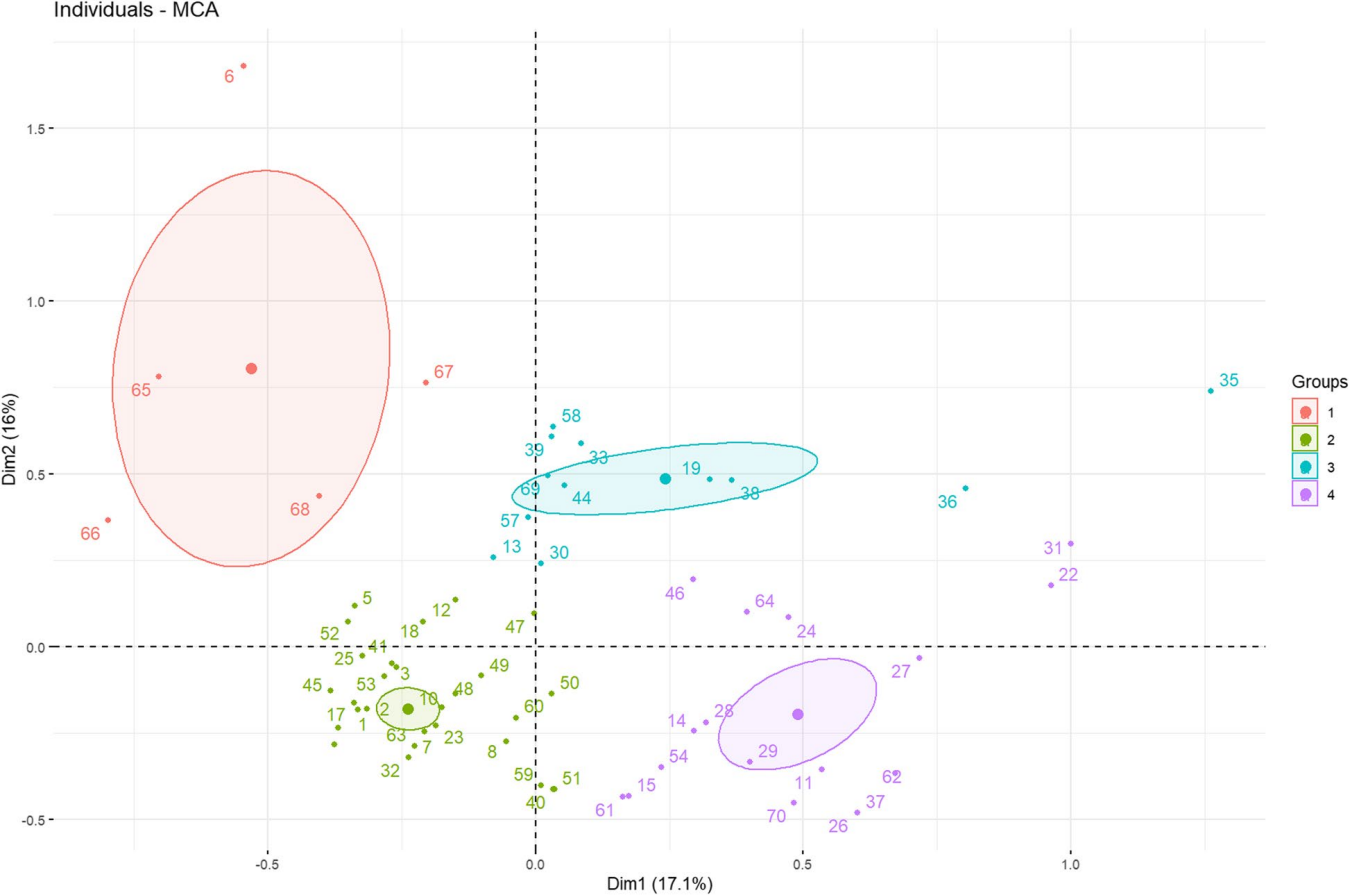
Biplot of both individuals and the values of the first two dimensions: Dim1 and Dim2. The center of each cluster has been highlighted

The partitioning algorithm suggested 4 as the optimal number of clusters. The first cluster is composed of 5 experts, the second of 36, the third 12 and the fourth includes 17 individuals. The resulting dendrogram is reported in Supplementary materials (Fig. S5). The bi-plot of individuals and the first two dimensions is shown in Fig. 2.

### Description of each cluster by quantitative variables (opinion variables)

**Fig. 3:**
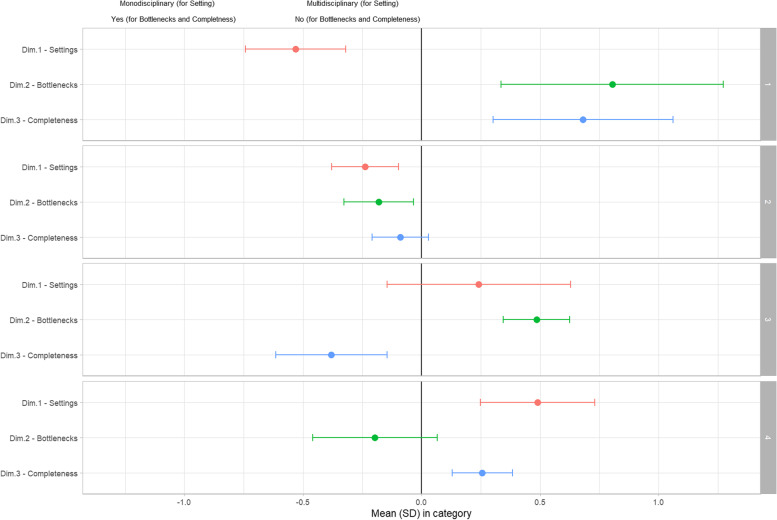
Dimensions in clusters: mean values with SD

A graphical representation of the clusters is shown in Fig. 3 with the resulting MCA dimensionvalues.

Cluster 1 has a mean Dim1 value of -0.54 ± 0.21, a mean Dim2 value of 0.80 ± 0.47 and a mean Dim3 value of 0.68 ± 0.38. Individuals belonging to this cluster strongly declare that the sub-processes are not complete (some activities are missing) and that the settings where they are currently executed are mono-disciplinary; moreover, they do not identify bottlenecks.

Cluster 2 has a mean Dim1 value of -0.24 ± 0.14, a mean Dim2 value of -0.18 ± 0.15 and a mean Dim3 value of -0.09 ± 0.12. Therefore, it comprises the experts whose opinion about the settings is that they are mono-disciplinary. They also think there are bottlenecks in the sub-processes and show a tendency towards a completeness judgment.

Cluster 3 has a mean Dim1 value of 0.24 ± 0.39, a mean Dim2 value of 0.49 ± 0.14 and a mean Dim3 value of -0.38 ± 0.24. The experts in this cluster do not see bottlenecks; moreover, they repute that the sub-processes are complete. The predominant opinion for the settings is that they are multidisciplinary.

Cluster 4 has a mean Dim1 value of 0.49 ± 0.24, a mean Dim2 value of -0.19 ± 0.26 and a mean Dim3 value of 0.26 ± 0.12. These individuals think the settings are multidisciplinary and they say the sub-processes are not complete. They tend to spot bottlenecks.

### Description of each cluster by categories of the characteristic variables

Table 2 reports the frequencies of the modalities regarding the characteristic variables in the total of the sample (Global %) and for each cluster. These findings are also graphically shown in the heat map reported in Fig. S6 in supplementary materials**.** Cluster 1 shows an overrepresentation of Japanese experts (40% of the cluster individuals—*p* = 0.024) that are currently working in a neuromuscular disease center (20%—*p* = 0.028). There is also a not significant tendency of working less frequently in a university center (20% versus a global modality of 58.6%—*p *= 0.1) but more often in a private center (60%—*p* = 0.15). In cluster 2 the Japanese provenience is not represented (0%—*p* = 0.051), and, most notably, these experts tend not to work in subspecialist settings. In fact, none of them worked in logopedic center (*p* = 0.051), occupational therapy center (*p* = 0.109), physiotherapy or rehabilitation center (*p* = 0.004). Cluster 3 seems not to show any distinct characteristic in comparison to the entire sample and therefore is the most representative of the global results. However, we noticed that it is not the largest cluster, with only 12 out of 70 experts (17%). The experts included in cluster 4 show a higher level of expertise, with 64.7% of them currently treating more than 20 NMD patients per month (*p* = 0.061, global 44.2%). None of them reported as working place a non-University center (*p* = 0.094). Remarkably, 94.1% (*p* = 0.04) worked in ambulatory as well as other specialist settings: logopedic center (17.65%—*p* = 0.044), occupational therapy center (11.76%—*p* = 0.157), physiotherapy or rehabilitation center (23.53%—*p* = 0.062). The expert typology (NMD neurologist or general neurologist) or the experience in NMD measured in years appear not to change significantly among the clusters.

**Table 2:** Predominant opinions and characteristics of the clusters. ** = *p* < 0.05 * = *p* < 0.1

	**Global %** **(*n* = 70)**	**Cl.1%** **(*n* = 5)**	**p**	**Cl.2%** **(*n* = 36)**	**p**	**Cl.3%** **(*n* = 12)**	**p**	**Cl.4%** **(*n* = 17)**	**p**
*Predominant opinion: Setting*		Mono		Mono		Multi		Multi	
*Predominant opinion: Bottleneck*		No		Yes		No		Yes	
*Predominant opinion: Completeness*		No		Yes		Yes		No	
*Region of origin: EU*	42.86	40	0.917	47.22	0.461	41.67	0.938	35.29	0.489
*Region of origin: JP*	5.71	**40**	0.024**	**0**	0.051*	8.33	0.671	5.88	0.927
*Region of origin: USA*	51.43	20	0.184	52.78	0.821	50	0.917	58.82	0.501
*Expert type: General Neurologist*	48.57	60	0.633	50	0.811	41.67	0.619	47.06	0.892
*Expert type: NMD Neurologist*	51.43	40	0.633	50	0.811	58.33	0.619	52.94	0.892
*Currently working in a NMD center*	70	**20**	0.028**	69.44	0.921	75	0.714	82.35	0.217
*N° of patients treated/month:* > *20*	44.29	40	0.868	38.89	0.363	33.33	0.427	**64.71**	0.061*
*N° of patients treated/month:* < *5*	7.14	20	0.355	8.33	0.724	8.33	0.822	0	0.237
*N° of patients treated/month: 5–20*	48.57	40	0.724	52.78	0.482	58.33	0.48	35.29	0.225
*Experience in NMD:* > *10 years*	70	60	0.63	63.89	0.267	75	0.714	82.35	0.217
*Experience in NMD:* < *5 years*	7.14	20	0.355	8.33	0.724	0	0.379	5.88	0.886
*Experience in NMD: 5—10 years*	22.86	20	0.941	27.78	0.332	25	0.828	11.76	0.229
*Working place: not for profit center*	2.86	0	0.861	0	0.232	8.33	0.343	5.88	0.486
*Working place: not University center*	11.43	0	0.535	16.67	0.182	16.67	0.547	**0**	0.094*
*Working place: private center*	28.57	60	0.158	22.22	0.242	25	0.799	35.29	0.494
*Working place: public center*	14.29	20	0.695	11.11	0.461	8.33	0.585	23.53	0.248
*Working place: University center*	58.57	20	0.1	63.89	0.367	58.33	0.978	58.82	0.988
*Working setting: hospital ambulatory*	75.71	100	0.237	69.44	0.225	58.33	0.155	**94.12**	0.04*
*Working setting: day-hospital*	24.29	0	0.237	22.22	0.69	33.33	0.443	29.41	0.579
*Working setting: hospital ward*	64.29	80	0.51	55.56	0.127	75	0.425	70.59	0.557
*Working setting: logopedic center*	5.71	20	0.285	**0**	0.051*	0	0.463	**17.65**	0.044**
*Working setting: occupational therapy center*	4.29	20	0.214	0	0.109	0	0.564	11.76	0.157
*Working setting: other*	1.43	0	0.929	2.78	0.514	0	0.829	0	0.757
*Working setting: physiotherapy / rehabilitation center*	10	20	0.494	**0**	0.004**	16.67	0.436	**23.53**	0.062*

## Discussion

The aim of this exploratory analysis was to analyze more in depth the expert opinion and its determinants when high quality evidence is lacking, such as when dealing with the development of a care pathway concerning a rare disease. From this cluster analysis some interesting results emerged: the first remarkable aspect is the poor representation of the not appropriateness variables in the factorial analysis. Even if the principal study highlighted a high level of agreement both on completeness and appropriateness (over 90%) [9], this may indicate a poor ability of the experts to discriminate what is not appropriate from what is not complete. In case of local implementation of a CP, this characteristic requires some consideration because it may lead to different outcomes: if the CP is not complete the main concern is that the patient may take unnecessary risks; conversely, if the CP contains not appropriate activities, the main issue is that the health care organization is wasting resources. Although both these aspects will ultimately have a negative impact on the patient health outcomes, the correctional measures to be employed are much different: where it may be easy to eliminate a not appropriate activity (at least in theory), it is not as easy to introduce a missing clinical activity. In fact, to add a missing activity in a CP would require a new round of validation among the experts. The cluster analysis clearly identified 4 different kinds of experts.

The expert from cluster 1 thinks that the care MG setting is multidisciplinary, does not spot bottlenecks and gives a completeness judgment. In this cluster most of the experts were not working in a NMD center. Moreover, the Japanese provenience is over-represented (40%): this may be due to the fact that there are few NMD centers in Japan, probably reflecting one of the smallest prevalence of MG as estimated in a recent study [16]. Lastly, the university workplace is underrepresented in favor of a higher frequency of private center working experts. Therefore, it seems that these experts have the least academic expertise of the whole panel, and their characteristics suggest that their tendency towards monodisciplinary settings might be related to a profession practiced predominantly in a private and not university workplace.

The second cluster main opinions include a mono-disciplinary setting with spotted bottlenecks and a completeness judgment of the sub-processes. The sub-specialist working settings in this cluster are clearly under-represented. We therefore reasonably think that, as the working setting shifts from sub specialist to hospital centered, the expert changes his opinion from not completeness to completeness, begins to spot bottlenecks, but he is still sure about the settings in which the sub-process are currently executed.

Cluster 3 is the most similar to the global results of the panel of experts. Even though some differences arise, none of them reached even quasi-significance, with the least p-value of 0.155 for the working setting of hospital ambulatory. Therefore, this cluster represents a sort of reference cluster, representative of the whole panel and no deduction nor speculation can be made on the basis of these results.

The cluster number 4 shows the predominant opinion on multidisciplinary settings, with subprocesses reputed not complete but with spotted bottlenecks. These experts are not working outside a university center (0%), often in sub-specialist settings. Given also that the experts treating more than 20 MG patients on a regularly monthly basis are over-represented, we can summarize this cluster as the one with the most expertise.

From the comparison among clusters, the first remarkable aspect is that, shifting from cluster 2 to cluster 4, as the working setting becomes more sub-specialistic, the opinions tend to move from a completeness judgement to a not completeness one. This may indicate a link between the working setting of the expert and his/her opinion about the completeness. Moreover, as expected, the opinion about the settings changes to multidisciplinary, probably reflecting the expert wide knowledge and experience of the settings. However, the opinion about bottlenecks appears not to be influenced by the working setting.

Interestingly, there are some characteristics that do not differ significantly among the clusters: the expert typology (whether general neurologist or NMD neurologist) and the experience in treating NMDs as measured in years. We think that the expert typology question may probe an opinion rather than a characteristic: since no official sub-specialty degree about NMD is currently recognized, the expert answered this question based on what he/she thought of himself/herself, rather than based on an own objective characteristic. The experience in NMD treatment measured in years, instead, appears not to be relevant as a determinant of the opinion of the experts in this field of rare diseases: we may speculate therefore that five years in treating this kind of patients is sufficient for a neurologist to be labeled as a NMD expert. This is different from what happens in other specialist medical fields such as the surgical ones, where practical experience clearly carries a major weight in defining a professional an “expert”.

### Strength of the study

The role of experience and other factors in the genesis of opinion is already demonstrated in other scientific fields of research such as climate change [17, 18], health determinants and social policy [19] or behavioral finance [20], and it remains a common subject of scientific study. Even in decision-making circumstances the opinion sometimes carries a high weight [21]. Despite this recognized importance and the already known difficulties to implement care pathway in local realities [22, 23], the expert opinion in this field of knowledge is still poorly investigated, even though it often provides the best evidence when dealing with rare diseases. To the author’s knowledge, this is the first attempt to analyze the expert opinion more in depth, given the fact that there is still an acknowledged need of its contribution in many medical specialties [24, 25].

### Limitations of the study

This study, being an exploratory analysis, has some major limitations and its results must be cautiously interpreted. Firstly, the analysis was conducted on a restricted panel of 70 MG experts. Therefore, we cannot be sure that these results are valid for the whole population of MG experts, or for diseases or conditions different from MG, or for any context different from the development of a care pathway. Secondly, the original questionnaire was designed for a different purpose study design; hence, to confirm these findings, a specific questionnaire and study must be created, validated, and ultimately conducted. Finally, as the opinions frequently change for many (even unknown) reasons, we cannot be sure that these findings will be still valid in the future. We did not plan a new round of validation of our results with the same experts because of legal reasons. However, we are considering to include in our further studies about CPs a specific analysis of the expert opinion which implies a validation of the results with a second round.

## Conclusion

The study of the determinants of the opinion when high quality evidence is scarce may be important to avoid incorporation of wrong concepts into guidelines, literature or, worse, CPs. Since the implementation of a CP must consider the local reality, to have deep knowledge of opinions may be of help in a successful implementation, and in reaching the quadruple aim of enhancing patient experience, improving population health, reducing costs and improving the work life of health care providers [26]. With this exploratory analysis we highlighted which characteristics of the experts might contribute to the genesis of their opinion. In brief, workplace and working settings seems to influence the opinions on completeness, settings and bottlenecks spotting of the MG care pathway sub-processes. On the contrary, such opinions might not be conditioned significantly by the provenience of the expert, years of experience or by the monthly volume of MG patients treated. Therefore, this study provides a rationale for developing more robust design studies to assess the relationship between the characteristics of the experts and their opinions, for a deeper understanding of the determinants of the opinion of clinical experts. Should these insights be confirmed, a recommendation of a more frequent interchange of MG experts among the NMD centers and among different sub-specialist settings might be made, because the more they experience different realities, the more accurate their opinions will be and the better their discrimination skills will be. Moreover, local implementer of pathways should be aware of the kind of local expert they are dealing with for a successful implementation because the opinions may also condition the resistance to organizational change, often required by the implementation process. Even if these insights may be of help in this aspect, further studies in this sense are strongly recommended. Finally, an overlap between non-appropriateness and completeness opinions might exist. More robust studies about this topic are recommended because a missing activity is much different from a non-appropriate activity both in terms of their consequences and in terms of correctional measures, and care pathway developers and local implementer must be aware of it.

## Supplementary Information

**Additional file 1. **Myasthenia Gravis Pathway - Complete Questionnaire.

**Additional file 2:** **Table S1.** Contribution of the “opinion” variables to the dimensions with the rank of the first ten variables. **Table S2. **Squared cosine (cos2 ) of the "opinion" variables. **Figure S1.** Scree plot of the results of MCA. **Figure S2.** Contribution of variables to dimension 1. **Figure S3.** Contribution of variables to dimension 2. **Figure S4.** Contribution of variables to dimension 3. **Figure S5.** HCPC resulting dendrogram. **Table S3.** Original output table from factomineR HCPC results with the assigned codes. **Table S4.** Extracted "Characteristic" questions with the recoded labels. **Table S5.** Extracted "Opinion" questions with the recoded labels (N.Ap = not appropriateness; BN = Bottlenecks; Set.= Setting; Cpl. = Completeness). **Figure S6.** Heatmap of the characteristic variables of the clusters of experts.

## Data Availability

The datasets used and/or analyzed during the current study available from the corresponding author on reasonable request.

## Abbreviations

CP: Care Pathway
MG: Myasthenia Gravis
MCA: Multiple correspondence analysis
HCPC: Hierarchical clustering on principal components
NMD: Neuromuscular disease
KI: Key interventions
