# Supplementary material for: The determinants of expert opinion in the development of care pathways: insights from an exploratory cluster analysis
Source: BMC Health Serv Res. 2023 Mar 3;23:211. doi: 10.1186/s12913-023-09139-7 (PMC9983158; doi:10.1186/s12913-023-09139-7)
Supplement: Supplementary file 1 — Additional file 1. Myasthenia Gravis Pathway - Complete Questionnaire. [file 12913_2023_9139_MOESM1_ESM.pdf]

Myasthenia Gravis Pathway - Complete Questionnaire

Legend
Q1-Q7 = characteristics of the expert
Q8-Q17 = Diagnosis subprocess
Q18-Q27 = Pharmacologic management subprocess
Q28-Q37 = Myastenic Crisis subprocess
Q38-Q47 = Speech&Swallow management subprocess
Q48-Q57 = Occupational /physiotherapy subprocess
Q58-Q67 = Psychological assessment and management subprocess
Q68-Q77 = Lifestyle factor assessment and management subprocess

Extracted question for secondary analysis are highlighted in yellow

	Question	Possible answers (if blank, the user could enter a detailed descriptive answer)
Q1	1.User ID (recoded as provenience from the first two letters of the code)	"-- Automatically compiled with an anonymous ID with the first two capital letters from the international code of the country of origin followed by 5 random digits --"
Q2	2.Q1 Please select your area of profession/expertise. (Important Note: This question is compulsory unless your preferred answer option is greyed out. In that case, you can leave the question unanswered and click on the Next button to submit)	Neuro-muscular expert (NMD) or General Neurologist
Q3	3.Are you working in Neuro-muscular disease expert centre?	Yes / No
Q4	4.Please select which type of institution are you working? Note: you can select more than one option.	Academic
		Non-academic
		Private
		Private not-for-profit
		Public
		Other
Q5	5.Please select the setting where you are working Note: you can select more than one option.	Out-patient clinic
		Day Hospital
		Hospital ward
		Occupational therapy centre
		Physio and rehabilitation centre
		Speech therapy centre
		Other
Q6	6.Please select the years of experience that you treat the MG patients	More than 10 years / Between 5 and 10 years / Less than 5 years
Q7	7.Please select the average number of MG patients you follow on a regular monthly base	More than 20 patients / Between 5 and 20 patients / Less than 5 patients
Q8	8.Based on your experience did you find any, not appropriate activity/intervention that was included in the above process flow?	Yes / No
Q9	9.Could you please flag the not appropriate ones from the following list.	Look for Muscle weakness with repetitive muscle use, symmetrically facial, neck, limb, truncal muscles involved or 4 mins of full-arm abduction or 20 mins abductions or speech becomes worse in some pts.
		Observe for Asymmetrical ptosis, the 5 mins of orbital cooling ice pack test demonstrates bilateral ptosis with >2mm ptosis: Functional ptosis could misdiagnose as MG but in this case eyebrow on the affected eye is lower than the unaffected eyebrow.
		Check whether MG muscle weakness can be immediately reversed by IV administration of fast-acting acetylcholinesterase inhibitors in patients with observable pareses.
		Perform Assays: anti-AChR and anti-MuSk anti-bodies.
		EMG repetitive stimulation.
		Single-fibre EMG.
		Acetylcholinesterase inhibitor test.
		Classify according to MGFA Clinical Classification
		Measure the outcomes by using instruments such as Quantified Myasthenia Gravis (QMG) Score, MG Quality of Life (MG-QOL 15), MG Manual Muscle Test (MG-MMT).
		Perform MRI or CT for Thymus screening.
Q10	10.Please analyze the below list of activities/interventions included in the subprocess (Diagnosis and assessment) that you examined before. Could you please rank the importance of those activities from 1 to 10 where 1 is the most important? (we realize that some activities will get similar importance but to prioritize them, you cannot attribute the same ranking for several activities). Note: To rank the activities you can either attribute the number directly in the box or drag them in the order you wish.	Look for Muscle weakness with repetitive muscle use, symmetrically facial, neck, limb, truncal muscles involved or 4 mins of full-arm abduction or 20 mins abductions or speech becomes worse in some pts.
		Observe for Asymmetrical ptosis, the 5 mins of orbital cooling ice pack test demonstrates bilateral ptosis with >2mm ptosis: Functional ptosis could misdiagnose as MG but in this case eyebrow on the affected eye is lower than the unaffected eyebrow.
		Check whether MG muscle weakness can be immediately reversed by IV administration of fast-acting acetylcholinesterase inhibitors in patients with observable pareses.
		Perform Assays: anti-AChR and anti-MuSk anti-bodies.
		EMG repetitive stimulation.
		Single-fibre EMG.
		Acetylcholinesterase inhibitor test.
		Classify according to MGFA Clinical Classification
		Measure the outcomes by using instruments such as Quantified Myasthenia Gravis (QMG) score, MG Quality of Life (MG-QOL 15), MG Manual Muscle Test (MG-MMT).
		Perform MRI or CT for Thymus screening.
Q11	11.Based on your experience, do you consider the above process flow complete?	Yes / No
Q12	12(a). Could you please specify which single most important activity/intervention is missing and include the level of evidence and the eventual reference.: Clinical activity/Intervention	
	12(b). Could you please specify which single most important activity/intervention is missing and include the level of evidence and the eventual reference.: Level of Evidence	
	12(c). Could you please specify which single most important activity/intervention is missing and include the level of evidence and the eventual reference.: Reference/Citation	
Q13	13.Based on your practical experience, could you please highlight the single most important potential bottleneck to the above process you may foresee as well at their frequency of occurrence? Example: After a hip fracture, clinical guidelines recommend immediate reparative surgery, within 24–48 hours from hospital admission. The organisational prerequisite when implementing this recommendation in real practice is the availability of operating room 24hrs seven days a week. In this case, several bottlenecks can affect this process: for example, during the summertime, it is likely that one hospital could reduce its activities because of the lack of personal for vacation. As a possible consequence, the trauma operating room will be open only five days a week from 8:00-20:00. In such a scenario if a patient is admitted for a hip fracture on Friday afternoon it will be impossible to be compliant with the recommendation. This is how an organisational bottleneck could affect the clinical process.	
Q14	14(a). What is the single most relevant outcome that can be influenced/determined by this part of the process of care? Please, indicate for each outcome its level of evidence (scientific literature and/or clinical expertise and/or your opinion).Example: After a hip fracture, clinical guidelines recommend immediate reparative surgery, within 24–48 hours from hospital admission. If the surgery is delayed, then there will be an increased risk of negative outcomes (patients death and pressure sores). : Outcome	
	14(b). What is the single most relevant outcome that can be influenced/determined by this part of the process of care? Please, indicate for each outcome its level of evidence (scientific literature and/or clinical expertise and/or your opinion).Example: After a hip fracture, clinical guidelines recommend immediate reparative surgery, within 24–48 hours from hospital admission. If the surgery is delayed, then there will be an increased risk of negative outcomes (patients death and pressure sores). : Level of Evidence	
	14(c). What is the single most relevant outcome that can be influenced/determined by this part of the process of care? Please, indicate for each outcome its level of evidence (scientific literature and/or clinical expertise and/or your opinion).Example: After a hip fracture, clinical guidelines recommend immediate reparative surgery, within 24–48 hours from hospital admission. If the surgery is delayed, then there will be an increased risk of negative outcomes (patients death and pressure sores). : Reference/Citation	
Q15	15(a). Could you please indicate the single most important typology/category of patients that should be excluded from this part of the process of MG care and the determining reason. Please also include the level of evidence of your answer (scientific literature and/or clinical expertise and/or your opinion). Example: After a hip fracture, clinical guidelines recommend immediate reparative surgery, within 24–48 hours from hospital admission. If the surgery is delayed, then there will be an increase in the risk of death and pressure sores. In this example, the patients with cardiac or renal failure, infections and uncontrolled diabetes or electrolyte abnormalities should be excluded, because they need to be visited cardiologists or nephrologists to evaluate the risk for surgery and often also require additional treatments and tests. This determines the impossibility to respect the appropriate timing of the operating room. Therefore, these patients cannot be included in the pathway.: Typology/category of patients should be excluded	
	15(b). Could you please indicate the single most important typology/category of patients that should be excluded from this part of the process of MG care and the determining reason. Please also include the level of evidence of your answer (scientific literature and/or clinical expertise and/or your opinion). Example: After a hip fracture, clinical guidelines recommend immediate reparative surgery, within 24–48 hours from hospital admission. If the surgery is delayed, then there will be an increase in the risk of death and pressure sores. In this example, the patients with cardiac or renal failure, infections and uncontrolled diabetes or electrolyte abnormalities should be excluded, because they need to be visited cardiologists or nephrologists to evaluate the risk for surgery and often also require additional treatments and tests. This determines the impossibility to respect the appropriate timing of the operating room. Therefore, these patients cannot be included in the pathway.: Reasons	
	15(c). Could you please indicate the single most important typology/category of patients that should be excluded from this part of the process of MG care and the determining reason. Please also include the level of evidence of your answer (scientific literature and/or clinical expertise and/or your opinion). Example: After a hip fracture, clinical guidelines recommend immediate reparative surgery, within 24–48 hours from hospital admission. If the surgery is delayed, then there will be an increase in the risk of death and pressure sores. In this example, the patients with cardiac or renal failure, infections and uncontrolled diabetes or electrolyte abnormalities should be excluded, because they need to be visited cardiologists or nephrologists to evaluate the risk for surgery and often also require additional treatments and tests. This determines the impossibility to respect the appropriate timing of the operating room. Therefore, these patients cannot be included in the pathway.: Level of evidence	

	15(d). Could you please indicate the single most important typology/category of patients that should be excluded from this part of the process of MG care and the determining reason. Please also include the level of evidence of your answer (scientific literature and/or clinical expertise and/or your opinion). Example: After a hip fracture, clinical guidelines recommend immediate reparative surgery, within 24–48 hours from hospital admission. If the surgery is delayed, then there will be an increase in the risk of death and pressure sores. In this example, the patients with cardiac or renal failure, infections and uncontrolled diabetes or electrolyte abnormalities should be excluded, because they need to be visited cardiologists or nephrologists to evaluate the risk for surgery and often also require additional treatments and tests. This determines the impossibility to respect the appropriate timing of the operating room. Therefore, these patients cannot be included in the pathway.: Reference/Citation	
Q16	16.According to your opinion, what is the most frequent setting where the above process is currently executed? (more than one answer allowed)	Ambulatory care
		Hospital day care
		Hospital in-patient
		Long-term care (nursing home)
		Long-term care (rehabilitation)
		Other
Q17	17.How is this list of activities/interventions is differing from the guidelines that you are using in your daily practice?	
Q18	18.Based on your experience did you find any, not appropriate activity/intervention that was included in the above process flow?	Yes / No
Q19	19.Could you please flag the not appropriate ones from the following list.	Patient with mild signs and symptoms: Start with acetylcholinesterase inhibitors.
		Check whether the patient has Changed in Bowel Pattern – Diarrhea during Acetylcholinesterase inhibitor treatment, if yes then 1.) Maintain a record of intake and output and number of stools. 2.) Monitor electrolyte studies Increase fluid intake. (Note: Carefully monitor Na and water retention in patients taking corticosteroids). 4.) Advance diet from liquid to bland to regular diet as tolerated. 5.) Instruct the patient to avoid foods such as fresh fruit, salads, or spicy or fried foods that may aggravate diarrhoea. 6.) Assess muscle strength to determine changes related to overdosage/under dosage of cholinesterase inhibitor medications. 7.) Instruct the patient to consult a physician for medications to control diarrhoea.
		If the patient does not respond with acetylcholinesterase inhibitors and the patient has Generalised MG (anti-AChR positive) consider thymectomy (if early onset of thymoma) and prescribe prednisone or prednisone plus azathioprine or mycophenolate mofetil.
		If the patient does not respond with acetylcholinesterase inhibitors and has Generalised MG (anti-MuSK positive) then prescribe prednisone or prednisone plus azathioprine or mycophenolate mofetil.
		If the treatment response is not good with prednisone or prednisone plus azathioprine or mycophenolate mofetil then switch to cyclosporin or tacrolimus or methotrexate, IVIg or subcutaneous immunoglobulin
		If the treatment response is not good with cyclosporin or tacrolimus or methotrexate, IVIg or subcutaneous immunoglobulin then switch to cyclophosphamide, rituximab or eculizumab, serial PE or IVIg
		If the patient does not respond to cyclophosphamide, rituximab or eculizumab, serial PE or IVIg. Then perform the re-evaluation of diagnosis
		After the re-evaluation of diagnosis, if the patient evaluated positive to anti-AChR or anti-MuSK then treat by reinstituting last effective dose, high or moderate dose prednisone, PE or IVIg if severe, Add or change immunosuppression.
		If the treatment response is not good with acetylcholinesterase inhibitors and the patient has only ocular MG then prescribe prednisone. If there is disappearance of the ocular signs and symptoms then continue treatment with lowest dose
		The patients who take prednisone need to be evaluated: a.) The Alteration in Fluid Balance Related to Na and H2O Retention. b.) Electrolyte Imbalance Related to Potassium Loss. c.) Weight Gain Related to Increased Appetite and Water Retention
		Patient who take prednisone and evaluated positive to; a.) Alteration in Fluid Balance Related to Na and H2O Retention. b.) Electrolyte Imbalance Related to Potassium Loss. c.) Weight Gain Related to Increased Appetite and Water Retention, then assist the patient with below interventions. 1.) Maintain a record of intake and output. 2.) Daily weights. 3.) Instruct patient on ways to decrease salt intake: – No added salt to food preparation. – No added salt to food at meal time. – Avoid salted snacks. 4.) Read food labels for sodium content. 5.) Avoid canned foods or frozen foods that add salt to food preparation. 6.) Monitor lab values and vital signs, and characteristics of pulse. 7.) Assess changes in muscle strength. 8.) Assist patient to include foods high in potassium, such as citrus fruits, green vegetables, meats, whole grain foods and fish. 9.) Instruct patient on principles of good nutrition. 10.) Instruct patients to avoid fad diets and to consult a physician before starting a weight loss program. 11.)Advise patient that fluctuations in appetite are a normal effect of steroid medications that require a conscious effort to control. 12.) Provide emotional support when changes in body image occur from weight gain from steroids.
		Patient with severe or moderate oropharyngeal signs/symptoms of MG, or respiratory crisis: Provide Supportive care, Bilevel positive-pressure ventilation, intubate if necessary, stop cholinesterase inhibitors and treat with PE or IVIg
Q20	20.Please have a look at the below list of activities/interventions included in the subprocess (MG assessment and pharmacological management) that you examined before. Could you please provide us with your opinion ranking the importance of those activities from 1 to 12 where 1 is the most important? (we realize that some activities will get similar importance but to prioritize them, you cannot attribute the same ranking for several activities). Note: To rank the activities you can either attribute the number directly in the box or drag them in the order you wish.	Patient with mild signs and symptoms: Start with acetylcholinesterase inhibitors.
		Check whether the patient has Changed in Bowel Pattern – Diarrhea during Acetylcholinesterase inhibitor treatment, if yes then 1.) Maintain a record of intake and output and number of stools. 2.) Monitor electrolyte studies Increase fluid intake. (Note: Carefully monitor Na and water retention in patients taking corticosteroids). 4.) Advance diet from liquid to bland to regular diet as tolerated. 5.) Instruct the patient to avoid foods such as fresh fruit, salads, or spicy or fried foods that may aggravate diarrhoea. 6.) Assess muscle strength to determine changes related to overdosage/under dosage of cholinesterase inhibitor medications. 7.) Instruct the patient to consult a physician for medications to control diarrhoea.
		If the patient does not respond with acetylcholinesterase inhibitors and the patient has Generalised MG (anti-AChR positive) consider thymectomy (if early onset of thymoma) and prescribe prednisone or prednisone plus azathioprine or mycophenolate mofetil.
		If the patient does not respond with acetylcholinesterase inhibitors and has Generalised MG (anti-MuSK positive) then prescribe prednisone or prednisone plus azathioprine or mycophenolate mofetil.
		If the treatment response is not good with prednisone or prednisone plus azathioprine or mycophenolate mofetil then switch to cyclosporin or tacrolimus or methotrexate, IVIg or subcutaneous immunoglobulin
		If the treatment response is not good with cyclosporin or tacrolimus or methotrexate, IVIg or subcutaneous immunoglobulin then switch to cyclophosphamide, rituximab or eculizumab, serial PE or IVIg
		If the patient does not respond to cyclophosphamide, rituximab or eculizumab, serial PE or IVIg. Then perform the re-evaluation of diagnosis
		After the re-evaluation of diagnosis, if the patient evaluated positive to anti-AChR or anti-MuSK then treat by reinstituting last effective dose, high or moderate dose prednisone, PE or IVIg if severe, Add or change immunosuppression.
		If the treatment response is not good with acetylcholinesterase inhibitors and the patient has only ocular MG then prescribe prednisone. If there is disappearance of the ocular signs and symptoms then continue treatment with lowest dose
		The patients who take prednisone need to be evaluated: a.) The Alteration in Fluid Balance Related to Na and H2O Retention. b.) Electrolyte Imbalance Related to Potassium Loss. c.) Weight Gain Related to Increased Appetite and Water Retention
		Patient who take prednisone and evaluated positive to; a.) Alteration in Fluid Balance Related to Na and H2O Retention. b.) Electrolyte Imbalance Related to Potassium Loss. c.) Weight Gain Related to Increased Appetite and Water Retention, then assist the patient with below interventions. 1.) Maintain a record of intake and output. 2.) Daily weights. 3.) Instruct patient on ways to decrease salt intake: – No added salt to food preparation. – No added salt to food at meal time. – Avoid salted snacks. 4.) Read food labels for sodium content. 5.) Avoid canned foods or frozen foods that add salt to food preparation. 6.) Monitor lab values and vital signs, and characteristics of pulse. 7.) Assess changes in muscle strength. 8.) Assist patient to include foods high in potassium, such as citrus fruits, green vegetables, meats, whole grain foods and fish. 9.) Instruct patient on principles of good nutrition. 10.) Instruct patients to avoid fad diets and to consult a physician before starting a weight loss program. 11.)Advise patient that fluctuations in appetite are a normal effect of steroid medications that require a conscious effort to control. 12.) Provide emotional support when changes in body image occur from weight gain from steroids.
		Patient with severe or moderate oropharyngeal signs/symptoms of MG, or respiratory crisis: Provide Supportive care, Bilevel positive-pressure ventilation, intubate if necessary, stop cholinesterase inhibitors and treat with PE or IVIg
Q21	21.Based on your experience, do you consider the above process flow complete?	Yes / No
Q22	22(a). Could you please specify in the below box which activity/intervention is missing (level of evidence and the eventual reference).: clinical activity/Intervention	
	22(b). Could you please specify in the below box which activity/intervention is missing (level of evidence and the eventual reference).: Level of Evidence	
	22(c). Could you please specify in the below box which activity/intervention is missing (level of evidence and the eventual reference).: Reference/Citation	
Q23	23.Based on your practical experience, could you please highlight the single most important potential bottleneck to the above process you may foresee as well at their frequency of occurrence? Example: After a hip fracture, clinical guidelines recommend immediate reparative surgery, within 24–48 hours from hospital admission. The organisational prerequisite when implementing this recommendation in real practice is the availability of operating room 24hrs seven days a week. In this case, several bottlenecks can affect this process: for example during the summertime, it is likely that one hospital could reduce its activities because of the lack of personal for vacation. As a possible consequence, the trauma operating room will be open only five days a week from 8:00-20:00. In such a scenario if a patient is admitted for a hip fracture on Friday afternoon it will be impossible to be compliant with the recommendation. This is how an organisational bottleneck could affect the clinical process.	
Q24	24(a). What is the single most relevant outcome that can be influenced/determined by this part of the process of care? Please, indicate for each outcome its level of evidence (scientific literature and/or clinical expertise and/or your opinion).Example: After a hip fracture, clinical guidelines recommend immediate reparative surgery, within 24–48 hours from hospital admission. If the surgery is delayed, then there will be an increased risk of negative outcomes (patients death and pressure sores). : Outcomes	
	24(b). What is the single most relevant outcome that can be influenced/determined by this part of the process of care? Please, indicate for each outcome its level of evidence (scientific literature and/or clinical expertise and/or your opinion).Example: After a hip fracture, clinical guidelines recommend immediate reparative surgery, within 24–48 hours from hospital admission. If the surgery is delayed, then there will be an increased risk of negative outcomes (patients death and pressure sores). : Level of evidence	

	24(c). What is the single most relevant outcome that can be influenced/determined by this part of the process of care? Please, indicate for each outcome its level of evidence (scientific literature and/or clinical expertise and/or your opinion).Example: After a hip fracture, clinical guidelines recommend immediate reparative surgery, within 24–48 hours from hospital admission. If the surgery is delayed, then there will be an increased risk of negative outcomes (patients death and pressure sores). : Reference/Citation	
Q25	25(a). Could you please indicate the single most important typology/category of patients that should be excluded from this part of the process of MG care and the determining reason. Please include also the level of evidence of your answer (scientific literature and/or clinical expertise and/or your opinion). Example: After a hip fracture, clinical guidelines recommend immediate reparative surgery, within 24–48 hours from hospital admission. If the surgery is delayed, then there will be an increase in the risk of death and pressure sores. In this example, the patients with cardiac or renal failure, infections and uncontrolled diabetes or electrolyte abnormalities should be excluded, because they need to be visited cardiologists or nephrologists to evaluate the risk for surgery and often also require additional treatments and tests. This determines the impossibility to respect the appropriate timing of the operating room. Therefore, these patients can not be included in the pathway.: Typology/category of patients should be excluded	
	25(b). Could you please indicate the single most important typology/category of patients that should be excluded from this part of the process of MG care and the determining reason. Please include also the level of evidence of your answer (scientific literature and/or clinical expertise and/or your opinion). Example: After a hip fracture, clinical guidelines recommend immediate reparative surgery, within 24–48 hours from hospital admission. If the surgery is delayed, then there will be an increase in the risk of death and pressure sores. In this example, the patients with cardiac or renal failure, infections and uncontrolled diabetes or electrolyte abnormalities should be excluded, because they need to be visited cardiologists or nephrologists to evaluate the risk for surgery and often also require additional treatments and tests. This determines the impossibility to respect the appropriate timing of the operating room. Therefore, these patients can not be included in the pathway.: Reasons	
	25(c). Could you please indicate the single most important typology/category of patients that should be excluded from this part of the process of MG care and the determining reason. Please include also the level of evidence of your answer (scientific literature and/or clinical expertise and/or your opinion). Example: After a hip fracture, clinical guidelines recommend immediate reparative surgery, within 24–48 hours from hospital admission. If the surgery is delayed, then there will be an increase in the risk of death and pressure sores. In this example, the patients with cardiac or renal failure, infections and uncontrolled diabetes or electrolyte abnormalities should be excluded, because they need to be visited cardiologists or nephrologists to evaluate the risk for surgery and often also require additional treatments and tests. This determines the impossibility to respect the appropriate timing of the operating room. Therefore, these patients can not be included in the pathway.: Level of evidence	
	25(d). Could you please indicate the single most important typology/category of patients that should be excluded from this part of the process of MG care and the determining reason. Please include also the level of evidence of your answer (scientific literature and/or clinical expertise and/or your opinion). Example: After a hip fracture, clinical guidelines recommend immediate reparative surgery, within 24–48 hours from hospital admission. If the surgery is delayed, then there will be an increase in the risk of death and pressure sores. In this example, the patients with cardiac or renal failure, infections and uncontrolled diabetes or electrolyte abnormalities should be excluded, because they need to be visited cardiologists or nephrologists to evaluate the risk for surgery and often also require additional treatments and tests. This determines the impossibility to respect the appropriate timing of the operating room. Therefore, these patients can not be included in the pathway.: Reference/Citation	
Q26	26.According to your opinion, what is the most frequent setting where the above process is currently executed? (more than one answer allowed)	Ambulatory care
		Hospital day care
		Hospital in-patient
		Long-term care (nursing home)
		Long-term care (rehabilitation)
		Other
Q27	27.How is this list of activities/interventions is differing from the guidelines that you are using in your daily practice?	
Q28	28.Based on your experience did you find any, not appropriate activity/intervention that was included in the above process flow?	Yes / No
Q29	29.Could you please flag the not appropriate ones from the following list.	Check for bulbar and respiratoryweakness
		Speech and Voice Assessment and Management: a.) If Oral intubation is necessary? - An intubation tube is passing through the pharynx, vocal folds and into the trachea and lungs, use an augmentative communication device. b.) If the patient's inability to communicate it is a failure to wean off the ventilator, necessitating a tracheostomy tube for prolonged ventilator use:- A ventilator Passy-Muir Tracheostomy Speaking Valve (vent valve) Or High-tech augmentative communication device is used.
		Treat the patients in intensive care with IVIg or plasma exchange plus treatment of infection and other precipitating events. If the patient responds, then Intensify long-term immune suppression.
		If the patient is not responding to IVIg or plasma exchange then prescribe plasma exchange or IVIg plus Glucocorticoids in high dose, treatment of infection and other precipitating events. If the patient responds, then Intensify long-term immune suppression.
		If the patient is not responding to plasma exchange or IVIg plus Glucocorticoids in high dose, prescribe other immunosuppressive drugs and treatment of complications. If the patient responds, then Intensify long-term immune suppression.
Q30	30.Please have a look at the below list of activities/interventions included in the subprocess (assessment and pharmacological management of MG crisis) that you examined before. Could you please provide us with your opinion ranking the importance of those activities from 1 to 5 where 1 is the most important? (we realize that some activities will get similar importance but to prioritize them, you cannot attribute the same ranking for several activities). Note: To rank the activities you can either attribute the number directly in the box or drag them in the order you wish.	Check for bulbar and respiratoryweakness
		Speech and Voice Assessment and Management: a.) If Oral intubation is necessary? - An intubation tube is passing through the pharynx, vocal folds and into the trachea and lungs, use an augmentative communication device. b.) If the patient's inability to communicate it is a failure to wean off the ventilator, necessitating a tracheostomy tube for prolonged ventilator use:- A ventilator Passy-Muir Tracheostomy Speaking Valve (vent valve) Or High-tech augmentative communication device is used.
		Treat the patients in intensive care with IVIg or plasma exchange plus treatment of infection and other precipitating events. If the patient responds, then Intensify long-term immune suppression.
		If the patient is not responding to IVIg or plasma exchange then prescribe plasma exchange or IVIg plus Glucocorticoids in high dose, treatment of infection and other precipitating events. If the patient responds, then Intensify long-term immune suppression.
		If the patient is not responding to plasma exchange or IVIg plus Glucocorticoids in high dose, prescribe other immunosuppressive drugs and treatment of complications. If the patient responds, then Intensify long-term immune suppression.
Q31	31.Based on your experience, do you consider the above process flow complete?	Yes / No
Q32	32(a). Could you please specify in the below box which activity/intervention is missing (level of evidence and the eventual reference).: clinical activity/Intervention	
	32(b). Could you please specify in the below box which activity/intervention is missing (level of evidence and the eventual reference).: Level of Evidence	
	32(c). Could you please specify in the below box which activity/intervention is missing (level of evidence and the eventual reference).: Reference/Citation	
Q33	33.Based on your practical experience, could you please highlight the single most important potential bottleneck to the above process you may foresee as well at their frequency of occurrence? Example: After a hip fracture, clinical guidelines recommend immediate reparative surgery, within 24–48 hours from hospital admission. The organisational prerequisite when implementing this recommendation in real practice is the availability of operating room 24hrs seven days a week. In this case, several bottlenecks can affect this process: for example during the summertime, it is likely that one hospital could reduce its activities because of the lack of personal for vacation. As a possible consequence, the trauma operating room will be open only five days a week from 8:00-20:00. In such a scenario if a patient is admitted for a hip fracture on Friday afternoon it will be impossible to be compliant with the recommendation. This is how an organisational bottleneck could affect the clinical process.	
Q34	34(a). What is the single most relevant outcome that can be influenced/determined by this part of the process of care? Please, indicate for each outcome its level of evidence (scientific literature and/or clinical expertise and/or your opinion).Example: After a hip fracture, clinical guidelines recommend immediate reparative surgery, within 24–48 hours from hospital admission. If the surgery is delayed, then there will be an increased risk of negative outcomes (patients death and pressure sores). : Outcomes	
	34(b). What is the single most relevant outcome that can be influenced/determined by this part of the process of care? Please, indicate for each outcome its level of evidence (scientific literature and/or clinical expertise and/or your opinion).Example: After a hip fracture, clinical guidelines recommend immediate reparative surgery, within 24–48 hours from hospital admission. If the surgery is delayed, then there will be an increased risk of negative outcomes (patients death and pressure sores). : Level of evidence	
	34(c). What is the single most relevant outcome that can be influenced/determined by this part of the process of care? Please, indicate for each outcome its level of evidence (scientific literature and/or clinical expertise and/or your opinion).Example: After a hip fracture, clinical guidelines recommend immediate reparative surgery, within 24–48 hours from hospital admission. If the surgery is delayed, then there will be an increased risk of negative outcomes (patients death and pressure sores). : Reference/Citation	
Q35	35(a). Could you please indicate the single most important typology/category of patients that should be excluded from this part of the process of MG care and the determining reason. Please include also the level of evidence of your answer (scientific literature and/or clinical expertise and/or your opinion). Example: After a hip fracture, clinical guidelines recommend immediate reparative surgery, within 24–48 hours from hospital admission. If the surgery is delayed, then there will be an increase in the risk of death and pressure sores. In this example, the patients with cardiac or renal failure, infections and uncontrolled diabetes or electrolyte abnormalities should be excluded, because they need to be visited cardiologists or nephrologists to evaluate the risk for surgery and often also require additional treatments and tests. This determines the impossibility to respect the appropriate timing of the operating room. Therefore, these patients can not be included in the pathway.: Typology/category of patients should be excluded	
	35(b). Could you please indicate the single most important typology/category of patients that should be excluded from this part of the process of MG care and the determining reason. Please include also the level of evidence of your answer (scientific literature and/or clinical expertise and/or your opinion). Example: After a hip fracture, clinical guidelines recommend immediate reparative surgery, within 24–48 hours from hospital admission. If the surgery is delayed, then there will be an increase in the risk of death and pressure sores. In this example, the patients with cardiac or renal failure, infections and uncontrolled diabetes or electrolyte abnormalities should be excluded, because they need to be visited cardiologists or nephrologists to evaluate the risk for surgery and often also require additional treatments and tests. This determines the impossibility to respect the appropriate timing of the operating room. Therefore, these patients can not be included in the pathway.: Reasons	

Q35	35(c). Could you please indicate the single most important typology/category of patients that should be excluded from this part of the process of MG care and the determining reason. Please include also the level of evidence of your answer (scientific literature and/or clinical expertise and/or your opinion). Example: After a hip fracture, clinical guidelines recommend immediate reparative surgery, within 24–48 hours from hospital admission. If the surgery is delayed, then there will be an increase in the risk of death and pressure sores. In this example, the patients with cardiac or renal failure, infections and uncontrolled diabetes or electrolyte abnormalities should be excluded, because they need to be visited cardiologists or nephrologists to evaluate the risk for surgery and often also require additional treatments and tests. This determines the impossibility to respect the appropriate timing of the operating room. Therefore, these patients can not be included in the pathway.: Level of evidence	
	35(d). Could you please indicate the single most important typology/category of patients that should be excluded from this part of the process of MG care and the determining reason. Please include also the level of evidence of your answer (scientific literature and/or clinical expertise and/or your opinion). Example: After a hip fracture, clinical guidelines recommend immediate reparative surgery, within 24–48 hours from hospital admission. If the surgery is delayed, then there will be an increase in the risk of death and pressure sores. In this example, the patients with cardiac or renal failure, infections and uncontrolled diabetes or electrolyte abnormalities should be excluded, because they need to be visited cardiologists or nephrologists to evaluate the risk for surgery and often also require additional treatments and tests. This determines the impossibility to respect the appropriate timing of the operating room. Therefore, these patients can not be included in the pathway.: Reference/Citation	
Q36	36.According to your opinion, what is the most frequent setting where the above process is currently executed? (more than one answer allowed)	Ambulatory care
		Hospital day care
		Hospital in-patient
		Long-term care (nursing home)
		Long-term care (rehabilitation)
		Other
Q37	37.How is this list of activities/interventions is differing from the guidelines that you are using in your daily practice?	
Q38	38.Based on your experience did you find any, not appropriate activity/intervention that was included in the above process flow?	Yes / No
Q39	39.Could you please flag the not appropriate ones from the following list.	Clinical swallowing examination: A.) Evaluate the musculature functioning of cranial nerves V, VII, IX, X, XI and XII is needed in all individuals with MG, especially patients with bulbar manifestations. B.)Test the fatigability of the facial, lingual and laryngeal functioning.
		Instrumental swallowing examination by using a Modified Barium swallow study (MBS)/ Fiberoptic Endoscopic evaluation of swallowing (FEES): A.) Evaluation of oropharyngeal and pharyngeal dysphagia. B.)View the hyolaryngeal elevation, tongue base to posterior pharyngeal wall approximation, pharyngeal contraction, upper esophageal sphincter (UES) opening and to perform a screen of esophageal motility by using MBS. C.) View the laryngeal physiology by using FEES. D.) Visualization of Pharyngeal Physiology. E.) Fatigability of Pharyngeal Functioning.
		Swallowing Rehabilitation: For improving one's oropharyngeal muscular baseline strength may decrease the degree of dysphagia.
		Lifetime Swallowing Plan: A.) Every three months assess swallowing in order to manage potential aspiration before it contributes to pulmonary complication and possible myasthenia crisis. B.) The speech-language pathologist (SLP) should make sure that patients and their caregivers educated to the signs and symptoms of dysphagia and aspiration. They should be knowledgeable and able to detect wet voice, throat clearing, coughing, increased chest congestion after eating, etc. C.) Patients should be instructed to periodically assess temperature and be alert to indicators of pneumonia.
		Compensatory Maneuvers: chin tuck, head turn or supraglottic swallow for eliminating or decreasing aspiration.
		Modify dental treatment to accommodate altered muscle strength: A.) Oral hygiene aids: 1.) Use Electric toothbrush. 2.) Use Manual toothbrush with a modified handle for ease of grasp. B.) Mouth prop, rubber dam isolation and saliva ejector use during dental treatment: 1.) Mouth props prevent muscle strain of having to hold the mouth open during treatment. 2.) Rubber dam isolation prevents worries about choking. By keeping dental materials and water spray out of the throat 3.) Saliva ejector suction tubing may be held and controlled by the patient to help avoid drooling and choking on saliva during procedures. C.) Dental chair position during Dental treatment: It is usually done in a reclining position. Let the dentist know if the patient is so far back that he feels like the throat is closing off. D.) Scheduled rest periods: Let the dentist know if the patient will need frequent rest breaks during treatment. E.) Compromised ability to manage complete dentures: 1.) The inability of the flaccid muscles to assist in retaining the lower denture and to maintain a peripheral seal for the upper denture. 2.) Overextended and over contoured maxillary dentures with thick flanges that impinge upon muscle and frenal attachments can lead to muscle fatigue and altered salivation. 3.) Ill-fitting dentures may exacerbate symptoms of difficulty in closing the mouth, tongue fatigue, a tight upper lip, dry mouth, impaired phonation, dysphagia and masticatory problems. F.) Monitor oral side effects/drug interactions of drugs/ therapies used to treat MG.
		Other
Q40	40.Please have a look at the below list of activities/interventions included in the subprocess (Speech, swallowing and dental functioning) that you examined before. Could you please provide us with your opinion ranking the importance of those activities from 1 to 6 where 1 is the most important? (we realize that some activities will get similar importance but to prioritize them, you cannot attribute the same ranking for several activities). Note: To rank the activities you can either attribute the number directly in the box or drag them in the order you wish.	Clinical swallowing examination: A.) Evaluate the musculature functioning of cranial nerves V, VII, IX, X, XI and XII is needed in all individuals with MG, especially patients with bulbar manifestations. B.)Test the fatigability of the facial, lingual and laryngeal functioning.
		Instrumental swallowing examination by using a Modified Barium swallow study (MBS)/ Fiberoptic Endoscopic evaluation of swallowing (FEES): A.) Evaluation of oropharyngeal and pharyngeal dysphagia. B.)View the hyolaryngeal elevation, tongue base to posterior pharyngeal wall approximation, pharyngeal contraction, upper esophageal sphincter (UES) opening and to perform a screen of esophageal motility by using MBS. C.) View the laryngeal physiology by using FEES. D.) Visualization of Pharyngeal Physiology. E.) Fatigability of Pharyngeal Functioning.
		Swallowing Rehabilitation: For improving one's oropharyngeal muscular baseline strength may decrease the degree of dysphagia.
		Lifetime Swallowing Plan: A.) Every three months assess swallowing in order to manage potential aspiration before it contributes to pulmonary complication and possible myasthenia crisis. B.) The speech-language pathologist (SLP) should make sure that patients and their caregivers educated to the signs and symptoms of dysphagia and aspiration. They should be knowledgeable and able to detect wet voice, throat clearing, coughing, increased chest congestion after eating, etc. C.) Patients should be instructed to periodically assess temperature and be alert to indicators of pneumonia.
		Compensatory Maneuvers: chin tuck, head turn or supraglottic swallow for eliminating or decreasing aspiration.
		Modify dental treatment to accommodate altered muscle strength: A.) Oral hygiene aids: 1.) Use Electric toothbrush. 2.) Use Manual toothbrush with a modified handle for ease of grasp. B.) Mouth prop, rubber dam isolation and saliva ejector use during dental treatment: 1.) Mouth props prevent muscle strain of having to hold the mouth open during treatment. 2.) Rubber dam isolation prevents worries about choking. By keeping dental materials and water spray out of the throat 3.) Saliva ejector suction tubing may be held and controlled by the patient to help avoid drooling and choking on saliva during procedures. C.) Dental chair position during Dental treatment: It is usually done in a reclining position. Let the dentist know if the patient is so far back that he feels like the throat is closing off. D.) Scheduled rest periods: Let the dentist know if the patient will need frequent rest breaks during treatment. E.) Compromised ability to manage complete dentures: 1.) The inability of the flaccid muscles to assist in retaining the lower denture and to maintain a peripheral seal for the upper denture. 2.) Overextended and over contoured maxillary dentures with thick flanges that impinge upon muscle and frenal attachments can lead to muscle fatigue and altered salivation. 3.) Ill-fitting dentures may exacerbate symptoms of difficulty in closing the mouth, tongue fatigue, a tight upper lip, dry mouth, impaired phonation, dysphagia and masticatory problems. F.) Monitor oral side effects/drug interactions of drugs/ therapies used to treat MG.
Q41	41.Based on your experience, do you consider the above process flow complete?	Yes / No
Q42	42(a). Could you please specify in the below box which activity/intervention is missing (level of evidence and the eventual reference).: clinical activity/Intervention	
	42(b). Could you please specify in the below box which activity/intervention is missing (level of evidence and the eventual reference).: Level of Evidence	
	42(c). Could you please specify in the below box which activity/intervention is missing (level of evidence and the eventual reference).: Reference/Citation	
Q43	43.Based on your practical experience, could you please highlight the single most important potential bottleneck to the above process you may foresee as well at their frequency of occurrence? Example: After a hip fracture, clinical guidelines recommend immediate reparative surgery, within 24–48 hours from hospital admission. The organisational prerequisite when implementing this recommendation in real practice is the availability of operating room 24hrs seven days a week. In this case, several bottlenecks can affect this process: for example during the summertime, it is likely that one hospital could reduce its activities because of the lack of personal for vacation. As a possible consequence, the trauma operating room will be open only five days a week from 8:00-20:00. In such a scenario if a patient is admitted for a hip fracture on Friday afternoon it will be impossible to be compliant with the recommendation. This is how an organisational bottleneck could affect the clinical process.	
Q44	44(a). What is the single most relevant outcome that can be influenced/determined by this part of the process of care? Please, indicate for each outcome its level of evidence (scientific literature and/or clinical expertise and/or your opinion).Example: After a hip fracture, clinical guidelines recommend immediate reparative surgery, within 24–48 hours from hospital admission. If the surgery is delayed, then there will be an increased risk of negative outcomes (patients death and pressure sores). : Outcomes	
	44(b). What is the single most relevant outcome that can be influenced/determined by this part of the process of care? Please, indicate for each outcome its level of evidence (scientific literature and/or clinical expertise and/or your opinion).Example: After a hip fracture, clinical guidelines recommend immediate reparative surgery, within 24–48 hours from hospital admission. If the surgery is delayed, then there will be an increased risk of negative outcomes (patients death and pressure sores). : Level of evidence	
	44(c). What is the single most relevant outcome that can be influenced/determined by this part of the process of care? Please, indicate for each outcome its level of evidence (scientific literature and/or clinical expertise and/or your opinion).Example: After a hip fracture, clinical guidelines recommend immediate reparative surgery, within 24–48 hours from hospital admission. If the surgery is delayed, then there will be an increased risk of negative outcomes (patients death and pressure sores). : Reference/Citation	
	45(a). Could you please indicate the single most important typology/category of patients that should be excluded from this part of the process of MG care and the determining reason. Please include also the level of evidence of your answer (scientific literature and/or clinical expertise and/or your opinion). Example: After a hip fracture, clinical guidelines recommend immediate reparative surgery, within 24–48 hours from hospital admission. If the surgery is delayed, then there will be an increase in the risk of death and pressure sores. In this example, the patients with cardiac or renal failure, infections and uncontrolled diabetes or electrolyte abnormalities should be excluded, because they need to be visited cardiologists or nephrologists to evaluate the risk for surgery and often also require additional treatments and tests. This determines the impossibility to respect the appropriate timing of the operating room. Therefore, these patients can not be included in the pathway.: Typology/category of patients should be excluded	

Q45	45(b). Could you please indicate the single most important typology/category of patients that should be excluded from this part of the process of MG care and the determining reason. Please include also the level of evidence of your answer (scientific literature and/or clinical expertise and/or your opinion). Example: After a hip fracture, clinical guidelines recommend immediate reparative surgery, within 24–48 hours from hospital admission. If the surgery is delayed, then there will be an increase in the risk of death and pressure sores. In this example, the patients with cardiac or renal failure, infections and uncontrolled diabetes or electrolyte abnormalities should be excluded, because they need to be visited cardiologists or nephrologists to evaluate the risk for surgery and often also require additional treatments and tests. This determines the impossibility to respect the appropriate timing of the operating room. Therefore, these patients can not be included in the pathway.: Reasons	
	45(c). Could you please indicate the single most important typology/category of patients that should be excluded from this part of the process of MG care and the determining reason. Please include also the level of evidence of your answer (scientific literature and/or clinical expertise and/or your opinion). Example: After a hip fracture, clinical guidelines recommend immediate reparative surgery, within 24–48 hours from hospital admission. If the surgery is delayed, then there will be an increase in the risk of death and pressure sores. In this example, the patients with cardiac or renal failure, infections and uncontrolled diabetes or electrolyte abnormalities should be excluded, because they need to be visited cardiologists or nephrologists to evaluate the risk for surgery and often also require additional treatments and tests. This determines the impossibility to respect the appropriate timing of the operating room. Therefore, these patients can not be included in the pathway.: Level of evidence	
	45(d). Could you please indicate the single most important typology/category of patients that should be excluded from this part of the process of MG care and the determining reason. Please include also the level of evidence of your answer (scientific literature and/or clinical expertise and/or your opinion). Example: After a hip fracture, clinical guidelines recommend immediate reparative surgery, within 24–48 hours from hospital admission. If the surgery is delayed, then there will be an increase in the risk of death and pressure sores. In this example, the patients with cardiac or renal failure, infections and uncontrolled diabetes or electrolyte abnormalities should be excluded, because they need to be visited cardiologists or nephrologists to evaluate the risk for surgery and often also require additional treatments and tests. This determines the impossibility to respect the appropriate timing of the operating room. Therefore, these patients can not be included in the pathway.: Reference/Citation	
Q46	46.According to your opinion, what is the most frequent setting where the above process is currently executed? (more than one answer allowed)	Ambulatory care<br>Hospital day care<br>Hospital in-patient<br>Long-term care (nursing home)<br>Long-term care (rehabilitation)<br>Other
Q47	47.How is this list of activities/interventions is differing from the guidelines that you are using in your daily practice?	
Q48	48.Based on your experience did you find any, not appropriate activity/intervention that was included in the above process flow?	Yes / No
Q49	49.Could you please flag the not appropriate ones from the following list.	Activities of Daily Living: 1.) Functional Independence Measures by using a.) Barthel Index. b.) Klein-Bell. 2.) Canadian Occupational Performance Measure. 3.) Myasthenia Gravis Activities of Daily Living Profile<br>Strength: Measure the Grip strength using dynamometry.<br>Fatigue: a.) Perceived Exertion Scale b.) Modified Fatigue Scale.<br>Vision/Ocular Motor Function: 1.) Extra Ocular Motility Exam. 2.) Hirschberg Light Reflex test. 3.) Maddox Rod test. 4.) Ptosis measurement in millimeters.<br>Instrumental Activities of Daily Living : 1.) Assessment of Motor and Process Skills. 2.) Canadian Occupational Performance Measure<br>Assessment of respiratory function: 1.) Check whether the patient is able to lie flat or bend over without shortness of breath 2.) Assess chest expansion and auscultate the chest in all quadrants for air entry or crackles. 3.) Observe for shortness of breath, increased respiratory effort or frequent inspiratory gasps. 4.) check for the rate, rhythm, and quality of respiration's and the presence of anxiety or restlessness. 5.) Oxygen saturation and blood gas analysis (additional).<br>Basic Self-Care: 1.) Use bathroom durable medical equipment to allow one to shower and groom while seated. 2.) Organize supplies and clothing such that minimal energy is spent obtaining items.<br>Home Management: 1.) Perform laundry in smaller loads. 2.) Use laundry hampers on casters that allow for ease in rolling rather than lifting. 3.) Organize frequently used cooking equipment/ utensils to allow ease in reaching and reduced trips to collect. 4.) Use lightweight equipment rather than heavy vacuum cleaners or mops.<br>Shopping: 1.) Plan shopping trips during the time of day that one has the most energy. 2.) Consider grocery stores that provide a "call-in" and pick-up service.<br>Parenting: Arrange diaper changing stations to allow performance while seated.<br>Care of Pets: 1.) Use elevated food container on casters. 2.) Use elevated feeding stations. 3.) Hire someone to take pets for a walk.<br>Leisure Activities: 1.) Perform more strenuous activities during peak medication response. 2.) Learn early signs of fatigue and allow sufficient recovery time. 3.) Balance strenuous activities with ones that require less physical exertion.<br>Sexual Activity: 1.) Plan sexual activities at times when concern for fatigue is not a factor. 2.) Utilize positions that require less energy for posture.
Q50	50.Please have a look at the below list of activities/interventions included in the subprocess (Occupational, Physical and Respiratory assessment and management) that you examined before. Could you please provide us with your opinion ranking the importance of those activities from 1 to 13 where 1 is the most important? (we realize that some activities will get similar importance but to prioritize them, you cannot attribute the same ranking for several activities). Note: To rank the activities you can either attribute the number directly in the box or drag them in the order you wish.	Activities of Daily Living: 1.) Functional Independence Measures by using a.) Barthel Index. b.) Klein-Bell. 2.) Canadian Occupational Performance Measure. 3.) Myasthenia Gravis Activities of Daily Living Profile<br>Strength: Measure the Grip strength using dynamometry.<br>Fatigue: a.) Perceived Exertion Scale b.) Modified Fatigue Scale.<br>Vision/Ocular Motor Function: 1.) Extra Ocular Motility Exam. 2.) Hirschberg Light Reflex test. 3.) Maddox Rod test. 4.) Ptosis measurement in millimeters.<br>Instrumental Activities of Daily Living : 1.) Assessment of Motor and Process Skills. 2.) Canadian Occupational Performance Measure<br>Assessment of respiratory function: 1.) Check whether the patient is able to lie flat or bend over without shortness of breath 2.) Assess chest expansion and auscultate the chest in all quadrants for air entry or crackles. 3.) Observe for shortness of breath, increased respiratory effort or frequent inspiratory gasps. 4.) check for the rate, rhythm, and quality of respiration's and the presence of anxiety or restlessness. 5.) Oxygen saturation and blood gas analysis (additional).<br>Basic Self-Care: 1.) Use bathroom durable medical equipment to allow one to shower and groom while seated. 2.) Organize supplies and clothing such that minimal energy is spent obtaining items.<br>Home Management: 1.) Perform laundry in smaller loads. 2.) Use laundry hampers on casters that allow for ease in rolling rather than lifting. 3.) Organize frequently used cooking equipment/ utensils to allow ease in reaching and reduced trips to collect. 4.) Use lightweight equipment rather than heavy vacuum cleaners or mops.<br>Shopping: 1.) Plan shopping trips during the time of day that one has the most energy. 2.) Consider grocery stores that provide a "call-in" and pick-up service.<br>Parenting: Arrange diaper changing stations to allow performance while seated.<br>Care of Pets: 1.) Use elevated food container on casters. 2.) Use elevated feeding stations. 3.) Hire someone to take pets for a walk.<br>Leisure Activities: 1.) Perform more strenuous activities during peak medication response. 2.) Learn early signs of fatigue and allow sufficient recovery time. 3.) Balance strenuous activities with ones that require less physical exertion.<br>Sexual Activity: 1.) Plan sexual activities at times when concern for fatigue is not a factor. 2.) Utilize positions that require less energy for posture.
Q51	51.Based on your experience, do you consider the above process flow complete?	Yes / No
Q52	52(a). Could you please specify in the below box which activity/intervention is missing (level of evidence and the eventual reference).: Clinical activity/Intervention	
	52(b). Could you please specify in the below box which activity/intervention is missing (level of evidence and the eventual reference).: Level of evidence	
	52(c). Could you please specify in the below box which activity/intervention is missing (level of evidence and the eventual reference).: Reference	
Q53	53.Based on your practical experience, could you please highlight the single most important potential bottleneck to the above process you may foresee as well at their frequency of occurrence? Example: After a hip fracture, clinical guidelines recommend immediate reparative surgery, within 24–48 hours from hospital admission. The organisational prerequisite when implementing this recommendation in real practice is the availability of operating room 24hrs seven days a week. In this case, several bottlenecks can affect this process: for example during the summertime, it is likely that one hospital could reduce its activities because of the lack of personal for vacation. As a possible consequence, the trauma operating room will be open only five days a week from 8:00-20:00. In such a scenario if a patient is admitted for a hip fracture on Friday afternoon it will be impossible to be compliant with the recommendation. This is how an organisational bottleneck could affect the clinical process.	
Q54	54(a). What is the single most relevant outcome that can be influenced/determined by this part of the process of care? Please, indicate for each outcome its level of evidence (scientific literature and/or clinical expertise and/or your opinion).Example: After a hip fracture, clinical guidelines recommend immediate reparative surgery, within 24–48 hours from hospital admission. If the surgery is delayed, then there will be an increased risk of negative outcomes (patients death and pressure sores). : Outcomes	
	54(b). What is the single most relevant outcome that can be influenced/determined by this part of the process of care? Please, indicate for each outcome its level of evidence (scientific literature and/or clinical expertise and/or your opinion).Example: After a hip fracture, clinical guidelines recommend immediate reparative surgery, within 24–48 hours from hospital admission. If the surgery is delayed, then there will be an increased risk of negative outcomes (patients death and pressure sores). : Level of evidence	
	54(c). What is the single most relevant outcome that can be influenced/determined by this part of the process of care? Please, indicate for each outcome its level of evidence (scientific literature and/or clinical expertise and/or your opinion).Example: After a hip fracture, clinical guidelines recommend immediate reparative surgery, within 24–48 hours from hospital admission. If the surgery is delayed, then there will be an increased risk of negative outcomes (patients death and pressure sores). : Reference/Citation	
	55(a). Could you please indicate the single most important typology/category of patients that should be excluded from this part of the process of MG care and the determining reason. Please include also the level of evidence of your answer (scientific literature and/or clinical expertise and/or your opinion). Example: After a hip fracture, clinical guidelines recommend immediate reparative surgery, within 24–48 hours from hospital admission. If the surgery is delayed, then there will be an increase in the risk of death and pressure sores. In this example, the patients with cardiac or renal failure, infections and uncontrolled diabetes or electrolyte abnormalities should be excluded, because they need to be visited cardiologists or nephrologists to evaluate the risk for surgery and often also require additional treatments and tests. This determines the impossibility to respect the appropriate timing of the operating room. Therefore, these patients can not be included in the pathway.: Typology/category of patients should be excluded	

Q55	55(b). Could you please indicate the single most important typology/category of patients that should be excluded from this part of the process of MG care and the determining reason. Please include also the level of evidence of your answer (scientific literature and/or clinical expertise and/or your opinion). Example: After a hip fracture, clinical guidelines recommend immediate reparative surgery, within 24–48 hours from hospital admission. If the surgery is delayed, then there will be an increase in the risk of death and pressure sores. In this example, the patients with cardiac or renal failure, infections and uncontrolled diabetes or electrolyte abnormalities should be excluded, because they need to be visited cardiologists or nephrologists to evaluate the risk for surgery and often also require additional treatments and tests. This determines the impossibility to respect the appropriate timing of the operating room. Therefore, these patients can not be included in the pathway.: Reasons	
	55(c). Could you please indicate the single most important typology/category of patients that should be excluded from this part of the process of MG care and the determining reason. Please include also the level of evidence of your answer (scientific literature and/or clinical expertise and/or your opinion). Example: After a hip fracture, clinical guidelines recommend immediate reparative surgery, within 24–48 hours from hospital admission. If the surgery is delayed, then there will be an increase in the risk of death and pressure sores. In this example, the patients with cardiac or renal failure, infections and uncontrolled diabetes or electrolyte abnormalities should be excluded, because they need to be visited cardiologists or nephrologists to evaluate the risk for surgery and often also require additional treatments and tests. This determines the impossibility to respect the appropriate timing of the operating room. Therefore, these patients can not be included in the pathway.: Level of evidence	
	55(d). Could you please indicate the single most important typology/category of patients that should be excluded from this part of the process of MG care and the determining reason. Please include also the level of evidence of your answer (scientific literature and/or clinical expertise and/or your opinion). Example: After a hip fracture, clinical guidelines recommend immediate reparative surgery, within 24–48 hours from hospital admission. If the surgery is delayed, then there will be an increase in the risk of death and pressure sores. In this example, the patients with cardiac or renal failure, infections and uncontrolled diabetes or electrolyte abnormalities should be excluded, because they need to be visited cardiologists or nephrologists to evaluate the risk for surgery and often also require additional treatments and tests. This determines the impossibility to respect the appropriate timing of the operating room. Therefore, these patients can not be included in the pathway.: Reference/Citation	
Q56	56.According to your opinion, what is the most frequent setting where the above process is currently executed? (more than one answer allowed)	Ambulatory care<br>Hospital day care<br>Hospital in-patient<br>Long-term care (nursing home)<br>Long-term care (rehabilitation)<br>Other
Q57	57.How is this list of activities/interventions is differing from the guidelines that you are using in your daily practice?	
Q58	58.Based on your experience did you find any, not appropriate activity/intervention that was included in the above process flow?	Yes / No
Q59	59.Could you please flag the not appropriate ones from the following list.	Assessment of Potentially dysfunctional self-concepts.<br>Assessment of Ineffective Coping Mechanisms.<br>Assessment of Altered role performance.<br>Assessment of Anxiety-related to the disease process and lifestyle alterations, Fear of death: patient family and friends.<br>Interventions for Potentially dysfunctional self-concepts: 1.) Encourage patients to verbalize the meaning of the illness/loss (i.e., "How do you feel about what is happening to you?") 2.) Listen attentively and compassionately. 3.) Since appearances may greatly alter and weakness may leave patients unable to take care of grooming needs, help them to look their best. 4.) Be honest about the realities of the illness; encourage patients to seek help if denial becomes detrimental. 5.) Facilitate acceptance; help patients set realistic, short-term goals so that success may be achieved. 6.) Encourage patients to do the things that they are capable of doing. 7.) Share hopeful aspects of the disease with patients and family. 8.) Recognize that the family too will be experiencing grief for the loss of the way the patient "used to be."<br>Interventions for Ineffective coping mechanism: 1.) Help patients to identify how they coped with adversity in the past<br>Interventions for Role performance strategies: 1.) Determine what their usual coping mechanisms are, and how they can best be used to cope with the MG. 2.) Assist patients in identifying factors in their environment that have the potential to undermine positive adaptation. 3.) Involve patients in planning and decision making regarding care. 4.) Give patients and family information regarding the disease, medications, emergency measures, and precautions for living with MG after discharge from the acute care setting. 5.) Explore patient role changes so that they will be less threatening 6.) Supply information on local MG chapter. Relationships can be formed with others with the disease and be a great source of strength to patients and family<br>Interventions for Anxiety-related to the disease process and Lifestyle alterations, Fear of death: patient family and friends 1.) Active Listening. 2.) Refer if necessary for counselling or therapy to deal with anxiety.
Q60	60.Please have a look at the below list of activities/interventions included in the subprocess (Psychological factors assessment and management) that you examined before. Could you please provide us with your opinion ranking the importance of those activities from 1 to 8 where 1 is the most important? (we realize that some activities will get similar importance but to prioritize them, you cannot attribute the same ranking for several activities). Note: To rank the activities you can either attribute the number directly in the box or drag them in the order you wish.	Assessment of Potentially dysfunctional self-concepts.<br>Assessment of Ineffective Coping Mechanisms.<br>Assessment of Altered role performance.<br>Assessment of Anxiety-related to the disease process and lifestyle alterations, Fear of death: patient family and friends.<br>Interventions for Potentially dysfunctional self-concepts: 1.) Encourage patients to verbalize the meaning of the illness/loss (i.e., "How do you feel about what is happening to you?") 2.) Listen attentively and compassionately. 3.) Since appearances may greatly alter and weakness may leave patients unable to take care of grooming needs, help them to look their best. 4.) Be honest about the realities of the illness; encourage patients to seek help if denial becomes detrimental. 5.) Facilitate acceptance; help patients set realistic, short-term goals so that success may be achieved. 6.) Encourage patients to do the things that they are capable of doing. 7.) Share hopeful aspects of the disease with patients and family. 8.) Recognize that the family too will be experiencing grief for the loss of the way the patient "used to be."<br>Interventions for Ineffective coping mechanism: 1.) Help patients to identify how they coped with adversity in the past<br>Interventions for Role performance strategies: 1.) Determine what their usual coping mechanisms are, and how they can best be used to cope with the MG. 2.) Assist patients in identifying factors in their environment that have the potential to undermine positive adaptation. 3.) Involve patients in planning and decision making regarding care. 4.) Give patients and family information regarding the disease, medications, emergency measures, and precautions for living with MG after discharge from the acute care setting. 5.) Explore patient role changes so that they will be less threatening 6.) Supply information on local MG chapter. Relationships can be formed with others with the disease and be a great source of strength to patients and family<br>Interventions for Anxiety-related to the disease process and Lifestyle alterations, Fear of death: patient family and friends 1.) Active Listening. 2.) Refer if necessary for counselling or therapy to deal with anxiety.
Q61	61.Based on your experience, do you consider the above process flow complete?	Yes / No
Q62	62(a). Could you please specify in the below box which activity/intervention is missing (level of evidence and the eventual reference).: clinical activity/Intervention	
	62(b). Could you please specify in the below box which activity/intervention is missing (level of evidence and the eventual reference).: Level of Evidence	
	62(c). Could you please specify in the below box which activity/intervention is missing (level of evidence and the eventual reference).: Reference/Citation	
Q63	63.Based on your practical experience, could you please highlight the single most important potential bottleneck to the above process you may foresee as well at their frequency of occurrence? Example: After a hip fracture, clinical guidelines recommend immediate reparative surgery, within 24–48 hours from hospital admission. The organisational prerequisite when implementing this recommendation in real practice is the availability of operating room 24hrs seven days a week. In this case, several bottlenecks can affect this process: for example during the summertime, it is likely that one hospital could reduce its activities because of the lack of personal for vacation. As a possible consequence, the trauma operating room will be open only five days a week from 8:00-20:00. In such a scenario if a patient is admitted for a hip fracture on Friday afternoon it will be impossible to be compliant with the recommendation. This is how an organisational bottleneck could affect the clinical process.	
Q64	64(a). What is the single most relevant outcome that can be influenced/determined by this part of the process of care? Please, indicate for each outcome its level of evidence (scientific literature and/or clinical expertise and/or your opinion).Example: After a hip fracture, clinical guidelines recommend immediate reparative surgery, within 24–48 hours from hospital admission. If the surgery is delayed, then there will be an increased risk of negative outcomes (patients death and pressure sores). : Outcomes	
	64(b). What is the single most relevant outcome that can be influenced/determined by this part of the process of care? Please, indicate for each outcome its level of evidence (scientific literature and/or clinical expertise and/or your opinion).Example: After a hip fracture, clinical guidelines recommend immediate reparative surgery, within 24–48 hours from hospital admission. If the surgery is delayed, then there will be an increased risk of negative outcomes (patients death and pressure sores). : Level of evidence	
	64(c). What is the single most relevant outcome that can be influenced/determined by this part of the process of care? Please, indicate for each outcome its level of evidence (scientific literature and/or clinical expertise and/or your opinion).Example: After a hip fracture, clinical guidelines recommend immediate reparative surgery, within 24–48 hours from hospital admission. If the surgery is delayed, then there will be an increased risk of negative outcomes (patients death and pressure sores). : Reference/Citation	
	65(a). Could you please indicate the single most important typology/category of patients that should be excluded from this part of the process of MG care and the determining reason. Please include also the level of evidence of your answer (scientific literature and/or clinical expertise and/or your opinion). Example: After a hip fracture, clinical guidelines recommend immediate reparative surgery, within 24–48 hours from hospital admission. If the surgery is delayed, then there will be an increase in the risk of death and pressure sores. In this example, the patients with cardiac or renal failure, infections and uncontrolled diabetes or electrolyte abnormalities should be excluded, because they need to be visited cardiologists or nephrologists to evaluate the risk for surgery and often also require additional treatments and tests. This determines the impossibility to respect the appropriate timing of the operating room. Therefore, these patients can not be included in the pathway.: Typology/category of patients should be excluded	

Q65	65(b). Could you please indicate the single most important typology/category of patients that should be excluded from this part of the process of MG care and the determining reason. Please include also the level of evidence of your answer (scientific literature and/or clinical expertise and/or your opinion). Example: After a hip fracture, clinical guidelines recommend immediate reparative surgery, within 24–48 hours from hospital admission. If the surgery is delayed, then there will be an increase in the risk of death and pressure sores. In this example, the patients with cardiac or renal failure, infections and uncontrolled diabetes or electrolyte abnormalities should be excluded, because they need to be visited cardiologists or nephrologists to evaluate the risk for surgery and often also require additional treatments and tests. This determines the impossibility to respect the appropriate timing of the operating room. Therefore, these patients can not be included in the pathway.: Reasons	
	65(c). Could you please indicate the single most important typology/category of patients that should be excluded from this part of the process of MG care and the determining reason. Please include also the level of evidence of your answer (scientific literature and/or clinical expertise and/or your opinion). Example: After a hip fracture, clinical guidelines recommend immediate reparative surgery, within 24–48 hours from hospital admission. If the surgery is delayed, then there will be an increase in the risk of death and pressure sores. In this example, the patients with cardiac or renal failure, infections and uncontrolled diabetes or electrolyte abnormalities should be excluded, because they need to be visited cardiologists or nephrologists to evaluate the risk for surgery and often also require additional treatments and tests. This determines the impossibility to respect the appropriate timing of the operating room. Therefore, these patients can not be included in the pathway.: Level of evidence	
	65(d). Could you please indicate the single most important typology/category of patients that should be excluded from this part of the process of MG care and the determining reason. Please include also the level of evidence of your answer (scientific literature and/or clinical expertise and/or your opinion). Example: After a hip fracture, clinical guidelines recommend immediate reparative surgery, within 24–48 hours from hospital admission. If the surgery is delayed, then there will be an increase in the risk of death and pressure sores. In this example, the patients with cardiac or renal failure, infections and uncontrolled diabetes or electrolyte abnormalities should be excluded, because they need to be visited cardiologists or nephrologists to evaluate the risk for surgery and often also require additional treatments and tests. This determines the impossibility to respect the appropriate timing of the operating room. Therefore, these patients can not be included in the pathway.: Reference/Citation	
Q66	66.According to your opinion, what is the most frequent setting where the above process is currently executed? (more than one answer allowed)	Ambulatory care
		Hospital day care
		Hospital in-patient
		Long-term care (nursing home)
		Long-term care (rehabilitation)
		Other
Q67	67.How is this list of activities/interventions is differing from the guidelines that you are using in your daily practice?	
Q68	68.Based on your experience did you find any, not appropriate activity/intervention that was included in the above process flow?	Yes / No
Q69	69.Could you please flag the not appropriate ones from the following list.	Nutritional assessment
		Stress and Coping Skills assessment
		Infection Control and Health Maintenance assessment
		Interventions for good Nutrition: 1.) Weight Control-low fat foods, lean protein, good carbohydrates and caloric intake based on age, gender, height, weight and activity level. 2.) Low Sodium -to reduce edema, breathing and other health problems. 3.) Potassium intake (bananas, avocado, broccoli, potato, citrus fruits, fish, dairy). Potassium depletion can occur due to corticosteroid treatment. 4.) Calcium (dairy, green leafy vegetables, broccoli, cauliflower, egg yolks, lentils, soft bones of salmon and sardines) for strong bones and teeth.
		Interventions for stress management and coping Skills: 1.) Avoid a sedentary lifestyle. 2.) Progressive relaxation, yoga, deep-breathing, physical activity (walking, stationary ergometer, weight training, treadmill, swimming), visualization, adequate rest and proper nutrition. 4.) Support groups, stress management programs, socialization and religious/spiritual activities. 5.) Psychological counselling.
		Interventions for infection control and health maintenance: 1.) Proper handwashing (20 seconds with soap and water, dry well, lotion on dry skin). 2.) Maintain good dental care and oral hygiene. 3.) Avoid large crowds and individuals with upper respiratory infections or other contagious illnesses. 4.) Keep current on immunizations as advised by the health care provider.
Q70	70.Please have a look at the below list of activities/interventions included in the subprocess (Life-style factors assessment and management) that you examined before. Could you please provide us with your opinion ranking the importance of those activities from 1 to 6 where 1 is the most important? (we realize that some activities will get similar importance but to prioritize them, you cannot attribute the same ranking for several activities). Note: To rank the activities you can either attribute the number directly in the box or drag them in the order you wish.	Nutritional assessment
		Stress and Coping Skills assessment
		Infection Control and Health Maintenance assessment
		Interventions for good Nutrition: 1.) Weight Control-low fat foods, lean protein, good carbohydrates and caloric intake based on age, gender, height, weight and activity level. 2.) Low Sodium -to reduce edema, breathing and other health problems. 3.) Potassium intake (bananas, avocado, broccoli, potato, citrus fruits, fish, dairy). Potassium depletion can occur due to corticosteroid treatment. 4.) Calcium (dairy, green leafy vegetables, broccoli, cauliflower, egg yolks, lentils, soft bones of salmon and sardines) for strong bones and teeth.
		Interventions for stress management and coping Skills: 1.) Avoid a sedentary lifestyle. 2.) Progressive relaxation, yoga, deep-breathing, physical activity (walking, stationary ergometer, weight training, treadmill, swimming), visualization, adequate rest and proper nutrition. 4.) Support groups, stress management programs, socialization and religious/spiritual activities. 5.) Psychological counselling.
		Interventions for infection control and health maintenance: 1.) Proper handwashing (20 seconds with soap and water, dry well, lotion on dry skin). 2.) Maintain good dental care and oral hygiene. 3.) Avoid large crowds and individuals with upper respiratory infections or other contagious illnesses. 4.) Keep current on immunizations as advised by the health care provider.
Q71	71.Based on your experience, do you consider the above process flow complete?	Yes / No
Q72	72(a). Could you please specify in the below box which activity/intervention is missing (level of evidence and the eventual reference).: clinical activity/Intervention	
	72(b). Could you please specify in the below box which activity/intervention is missing (level of evidence and the eventual reference).: Level of Evidence	
	72(c). Could you please specify in the below box which activity/intervention is missing (level of evidence and the eventual reference).: Reference/Citation	
Q73	73.Based on your practical experience, could you please highlight the single most important potential bottleneck to the above process you may foresee as well at their frequency of occurrence? Example: After a hip fracture, clinical guidelines recommend immediate reparative surgery, within 24–48 hours from hospital admission. The organisational prerequisite when implementing this recommendation in real practice is the availability of operating room 24hrs seven days a week. In this case, several bottlenecks can affect this process: for example during the summertime, it is likely that one hospital could reduce its activities because of the lack of personal for vacation. As a possible consequence, the trauma operating room will be open only five days a week from 8:00-20:00. In such a scenario if a patient is admitted for a hip fracture on Friday afternoon it will be impossible to be compliant with the recommendation. This is how an organisational bottleneck could affect the clinical process.	
Q74	74(a). What is the single most relevant outcome that can be influenced/determined by this part of the process of care? Please, indicate for each outcome its level of evidence (scientific literature and/or clinical expertise and/or your opinion).Example: After a hip fracture, clinical guidelines recommend immediate reparative surgery, within 24–48 hours from hospital admission. If the surgery is delayed, then there will be an increased risk of negative outcomes (patients death and pressure sores). : Outcomes	
	74(b). What is the single most relevant outcome that can be influenced/determined by this part of the process of care? Please, indicate for each outcome its level of evidence (scientific literature and/or clinical expertise and/or your opinion).Example: After a hip fracture, clinical guidelines recommend immediate reparative surgery, within 24–48 hours from hospital admission. If the surgery is delayed, then there will be an increased risk of negative outcomes (patients death and pressure sores). : Level of evidence	
	74(c). What is the single most relevant outcome that can be influenced/determined by this part of the process of care? Please, indicate for each outcome its level of evidence (scientific literature and/or clinical expertise and/or your opinion).Example: After a hip fracture, clinical guidelines recommend immediate reparative surgery, within 24–48 hours from hospital admission. If the surgery is delayed, then there will be an increased risk of negative outcomes (patients death and pressure sores). : Reference/Citation	
Q75	75(a). Could you please indicate the single most important typology/category of patients that should be excluded from this part of the process of MG care and the determining reason. Please include also the level of evidence of your answer (scientific literature and/or clinical expertise and/or your opinion). Example: After a hip fracture, clinical guidelines recommend immediate reparative surgery, within 24–48 hours from hospital admission. If the surgery is delayed, then there will be an increase in the risk of death and pressure sores. In this example, the patients with cardiac or renal failure, infections and uncontrolled diabetes or electrolyte abnormalities should be excluded, because they need to be visited cardiologists or nephrologists to evaluate the risk for surgery and often also require additional treatments and tests. This determines the impossibility to respect the appropriate timing of the operating room. Therefore, these patients can not be included in the pathway.: Typology/category of patients should be excluded	
	75(b). Could you please indicate the single most important typology/category of patients that should be excluded from this part of the process of MG care and the determining reason. Please include also the level of evidence of your answer (scientific literature and/or clinical expertise and/or your opinion). Example: After a hip fracture, clinical guidelines recommend immediate reparative surgery, within 24–48 hours from hospital admission. If the surgery is delayed, then there will be an increase in the risk of death and pressure sores. In this example, the patients with cardiac or renal failure, infections and uncontrolled diabetes or electrolyte abnormalities should be excluded, because they need to be visited cardiologists or nephrologists to evaluate the risk for surgery and often also require additional treatments and tests. This determines the impossibility to respect the appropriate timing of the operating room. Therefore, these patients can not be included in the pathway.: Reasons	
	75(c). Could you please indicate the single most important typology/category of patients that should be excluded from this part of the process of MG care and the determining reason. Please include also the level of evidence of your answer (scientific literature and/or clinical expertise and/or your opinion). Example: After a hip fracture, clinical guidelines recommend immediate reparative surgery, within 24–48 hours from hospital admission. If the surgery is delayed, then there will be an increase in the risk of death and pressure sores. In this example, the patients with cardiac or renal failure, infections and uncontrolled diabetes or electrolyte abnormalities should be excluded, because they need to be visited cardiologists or nephrologists to evaluate the risk for surgery and often also require additional treatments and tests. This determines the impossibility to respect the appropriate timing of the operating room. Therefore, these patients can not be included in the pathway.: Level of evidence	

	75(d). Could you please indicate the single most important typology/category of patients that should be excluded from this part of the process of MG care and the determining reason. Please include also the level of evidence of your answer (scientific literature and/or clinical expertise and/or your opinion). Example: After a hip fracture, clinical guidelines recommend immediate reparative surgery, within 24–48 hours from hospital admission. If the surgery is delayed, then there will be an increase in the risk of death and pressure sores. In this example, the patients with cardiac or renal failure, infections and uncontrolled diabetes or electrolyte abnormalities should be excluded, because they need to be visited cardiologists or nephrologists to evaluate the risk for surgery and often also require additional treatments and tests. This determines the impossibility to respect the appropriate timing of the operating room. Therefore, these patients can not be included in the pathway.: Reference/Citation	
Q76	76.According to your opinion, what is the most frequent setting where the above process is currently executed? (more than one answer allowed)	Ambulatory care<br>Hospital day care<br>Hospital in-patient<br>Long-term care (nursing home)<br>Long-term care (rehabilitation)<br>Other
Q77	77.How is this list of activities/interventions is differing from the guidelines that you are using in your daily practice?	
