# Supplementary material for: The determinants of expert opinion in the development of care pathways: insights from an exploratory cluster analysis
Source: BMC Health Serv Res. 2023 Mar 3;23:211. doi: 10.1186/s12913-023-09139-7 (PMC9983158; doi:10.1186/s12913-023-09139-7)

Supplementary materials

The determinants of expert opinion in the care pathway development: insights from an exploratory cluster analysis.

Table S1 - Contribution of the "opinion" variables to the dimensions with the rank of the first ten variables

	Dim1 (var %)	Dim1 Rank	Dim2 (var %)	Dim2 Rank	Dim3 (var %)	Dim3 Rank
**N.Ap.1_Yes**	0.4659		1.4198		0.5948	
**N.Ap.1_No**	0.0518		0.1578		0.0661	
**N.Ap.2_Yes**	0.2718		4.0800		1.0565	
**N.Ap.2_No**	0.0351		0.5264		0.1363	
**N.Ap.3_Yes**	0.3075		1.1952		4.8405	8
**N.Ap.3_No**	0.0288		0.1120		0.4538	
**N.Ap.4_Yes**	0.0602		2.1354		0.0847	
**N.Ap.4_No**	0.0056		0.2002		0.0079	
**N.Ap.5_Yes**	0.0091		0.3448		3.2584	10
**N.Ap.5_No**	0.0006		0.0209		0.1975	
**N.Ap.6_Yes**	2.0800		2.8546		5.7270	6
**N.Ap.6_No**	0.1261		0.1730		0.3471	
**N.Ap.7_Yes**	0.9084		1.7389		4.9483	7
**N.Ap.7_No**	0.0852		0.1630		0.4639	
**Cpl.1_Yes**	0.0059		0.0060		0.0057	
**Cpl.1_No**	0.4088		0.4109		0.3929	
**Cpl.2_Yes**	0.0255		0.3316		0.4013	
**Cpl.2_No**	0.4216		5.4720	3	6.6222	5
**Cpl.3_Yes**	0.0099		0.4372		0.3427	
**Cpl.3_No**	0.1055		4.6630	7	3.6551	9
**Cpl.4_Yes**	0.0659		0.4570		0.6067	
**Cpl.4_No**	0.8570		5.9407	2	7.8866	4
**Cpl.5_Yes**	0.0799		0.1838		0.4224	
**Cpl.5_No**	1.7836		4.1057		9.4347	3
**Cpl.6_Yes**	0.1095		0.2384		0.5208	
**Cpl.6_No**	2.4455		5.3254	5	11.6302	1
**Cpl.7_Yes**	0.0465		0.1231		0.2945	
**Cpl.7_No**	1.5807		4.1844		10.0131	2
**BN.1_No**	0.4064		4.3389	8	0.0871	
**BN.1_Yes**	0.0677		0.7231		0.0145	
**BN.2_No**	1.5414		5.0513	6	0.1990	
**BN.2_Yes**	0.4204		1.3776		0.0543	
**BN.3_No**	0.8108		4.2378	9	1.5897	
**BN.3_Yes**	0.3243		1.6951		0.6359	
**BN.4_No**	0.9904		3.8802		1.9667	
**BN.4_Yes**	0.4245		1.6629		0.8429	
**BN.5_No**	1.4713		4.2137	10	0.4929	
**BN.5_Yes**	1.1695		3.3494		0.3918	
**BN.6_No**	2.0531		5.4194	4	0.1303	

**BN.6_Yes**	1.2132		3.2024		0.0770	
**BN.7_No**	2.1282		6.2757	1	1.2548	
**BN.7_Yes**	0.9754		2.8763		0.5751	
**Set.1_mono**	1.2842		0.0044		0.3706	
**Set.1_multi**	6.2071	5	0.0211		1.7914	
**Set.2_mono**	1.4580		0.0429		0.3698	
**Set.2_multi**	5.8318	7	0.1715		1.4790	
**Set.3_mono**	2.5476		0.0026		0.2151	
**Set.3_multi**	8.5980	3	0.0089		0.7260	
**Set.4_mono**	4.4499	8	0.2933		1.3173	
**Set.4_multi**	8.0098	4	0.5280		2.3711	
**Set.5_mono**	3.3815		0.1679		0.5862	
**Set.5_multi**	9.0767	2	0.4507		1.5734	
**Set.6_mono**	3.7841	9	0.1896		1.0155	
**Set.6_multi**	9.4602	1	0.4739		2.5388	
**Set.7_mono**	3.6769	10	0.9025		1.1271	
**Set.7_multi**	5.8557	6	1.4373		1.7950	

Table S2 - Squared cosine (cos^2) of the "opinion" variables

	**Dim1**	**Dim2**	**Dim3**
**N.Ap.1_Yes**	0.0247	0.0705	0.0201
**N.Ap.1_No**	0.0247	0.0705	0.0201
**N.Ap.2_Yes**	0.0147	0.2057	0.0364
**N.Ap.2_No**	0.0147	0.2057	0.0364
**N.Ap.3_Yes**	0.0161	0.0584	0.1614
**N.Ap.3_No**	0.0161	0.0584	0.1614
**N.Ap.4_Yes**	0.0031	0.1043	0.0028
**N.Ap.4_No**	0.0031	0.1043	0.0028
**N.Ap.5_Yes**	0.0005	0.0163	0.1054
**N.Ap.5_No**	0.0005	0.0163	0.1054
**N.Ap.6_Yes**	0.1054	0.1352	0.1852
**N.Ap.6_No**	0.1054	0.1352	0.1852
**N.Ap.7_Yes**	0.0475	0.0850	0.1650
**N.Ap.7_No**	0.0475	0.0850	0.1650
**Cpl.1_Yes**	0.0198	0.0186	0.0122
**Cpl.1_No**	0.0198	0.0186	0.0122
**Cpl.2_Yes**	0.0214	0.2592	0.2141
**Cpl.2_No**	0.0214	0.2592	0.2141
**Cpl.3_Yes**	0.0055	0.2278	0.1219
**Cpl.3_No**	0.0055	0.2278	0.1219
**Cpl.4_Yes**	0.0441	0.2858	0.2589
**Cpl.4_No**	0.0441	0.2858	0.2589
**Cpl.5_Yes**	0.0890	0.1916	0.3005
**Cpl.5_No**	0.0890	0.1916	0.3005
**Cpl.6_Yes**	0.1220	0.2485	0.3704
**Cpl.6_No**	0.1220	0.2485	0.3704
**Cpl.7_Yes**	0.0777	0.1924	0.3142

**Cpl.7_No**	0.0777	0.1924	0.3142
**BN.1_No**	0.0226	0.2261	0.0031
**BN.1_Yes**	0.0226	0.2261	0.0031
**BN.2_No**	0.0937	0.2871	0.0077
**BN.2_Yes**	0.0937	0.2871	0.0077
**BN.3_No**	0.0542	0.2650	0.0678
**BN.3_Yes**	0.0542	0.2650	0.0678
**BN.4_No**	0.0676	0.2476	0.0857
**BN.4_Yes**	0.0676	0.2476	0.0857
**BN.5_No**	0.1261	0.3378	0.0270
**BN.5_Yes**	0.1261	0.3378	0.0270
**BN.6_No**	0.1560	0.3851	0.0063
**BN.6_Yes**	0.1560	0.3851	0.0063
**BN.7_No**	0.1482	0.4088	0.0558
**BN.7_Yes**	0.1482	0.4088	0.0558
**Set.1_mono**	0.3578	0.0011	0.0659
**Set.1_multi**	0.3578	0.0011	0.0659
**Set.2_mono**	0.3481	0.0096	0.0564
**Set.2_multi**	0.3481	0.0096	0.0564
**Set.3_mono**	0.5323	0.0005	0.0287
**Set.3_multi**	0.5323	0.0005	0.0287
**Set.4_mono**	0.5950	0.0367	0.1124
**Set.4_multi**	0.5950	0.0367	0.1124
**Set.5_mono**	0.5950	0.0276	0.0658
**Set.5_multi**	0.5950	0.0276	0.0658
**Set.6_mono**	0.6325	0.0296	0.1084
**Set.6_multi**	0.6325	0.0296	0.1084
**Set.7_mono**	0.4552	0.1045	0.0891
**Set.7_multi**	0.4552	0.1045	0.0891

Figure S1 - Scree plot of the results of MCA

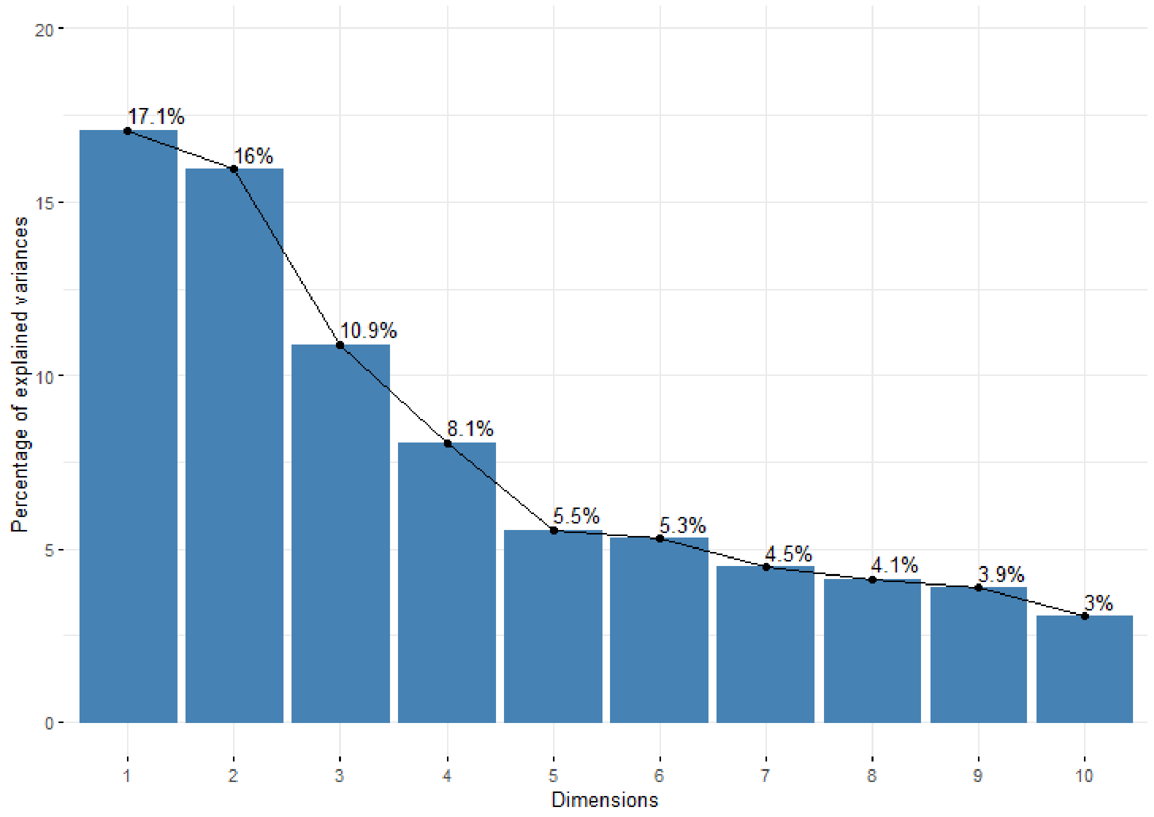

Figure S2 - Contribution of variables to dimension 1

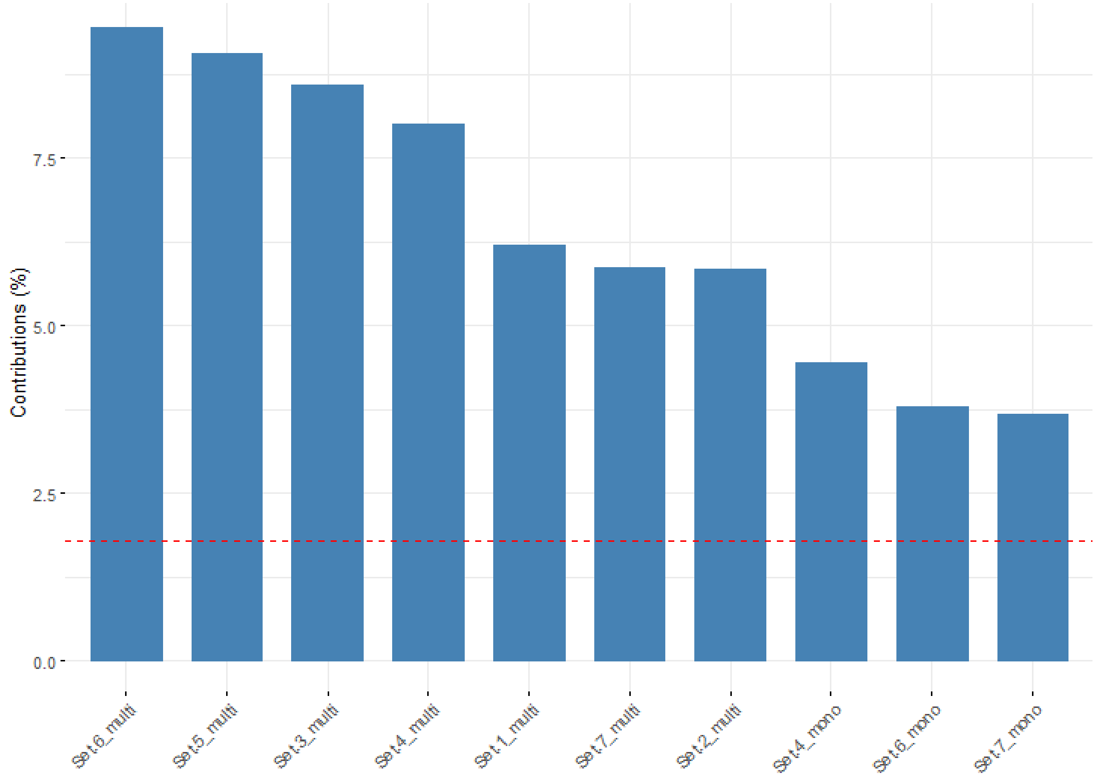

Figure S3 - Contribution of variables to dimension 2

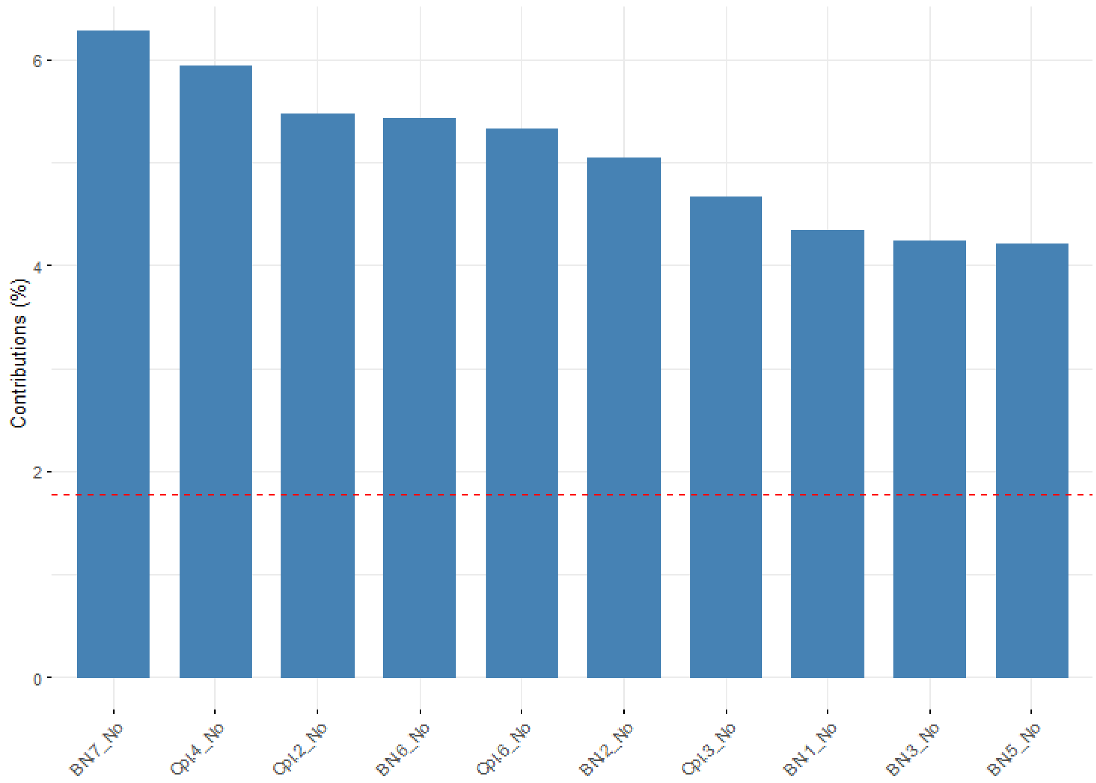

Figure S4 - Contribution of variables to dimension 3

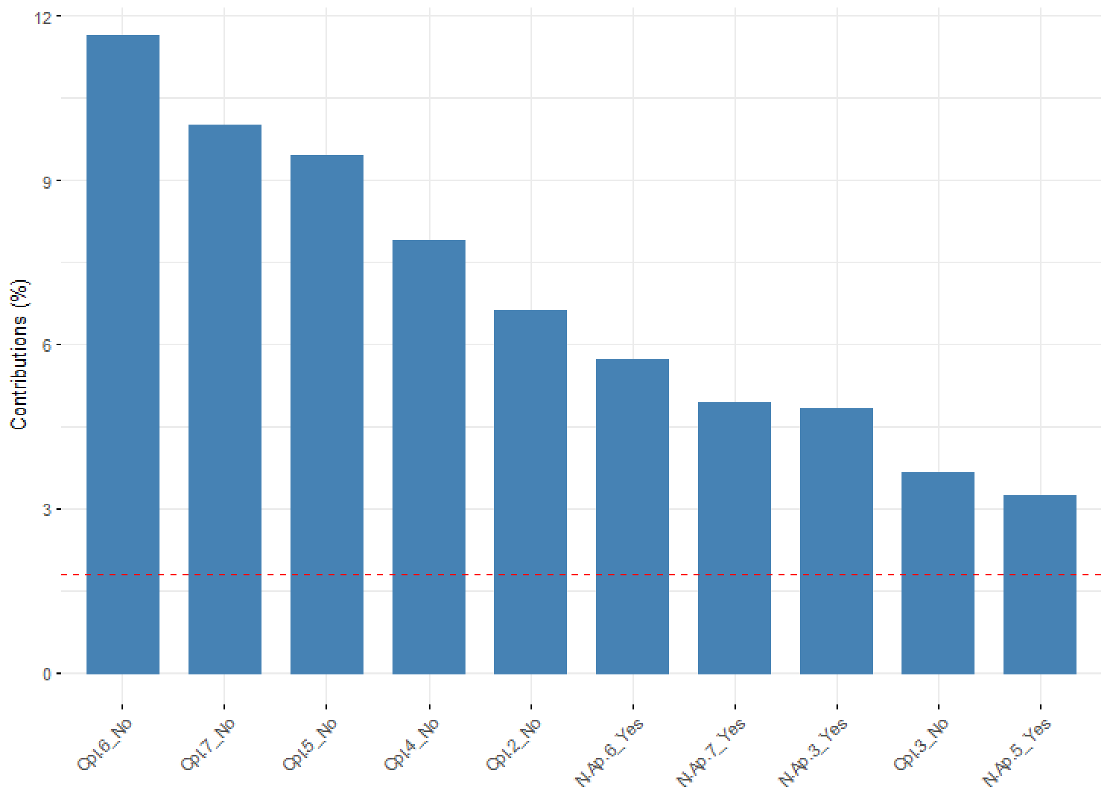

Figure S5 - HCPC resulting dendrogram

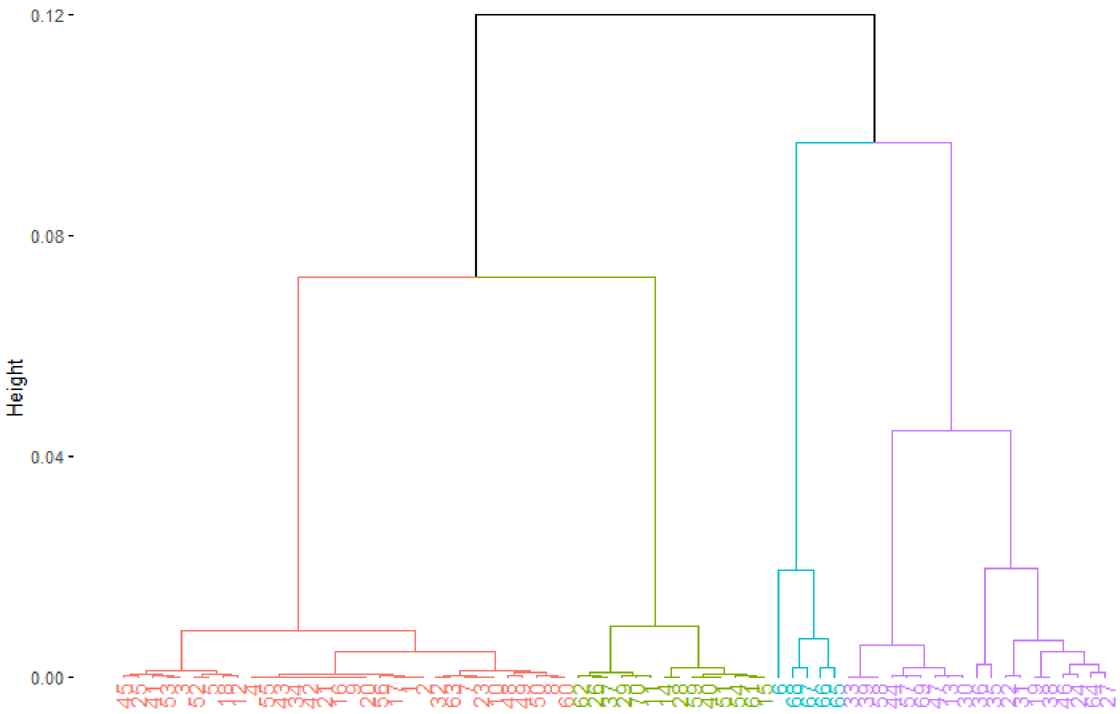

Table S3 - Original output table from factomineR HCPC results with the assigned codes

name	**Global**	**Mod/C 1**	**p**	**Mod/C 2**	**p**	**Mod/C 3**	**p**	**Mod/C 4**	**p**
Ctry=EU	42.86	40	0.917	47.22	0.461	41.67	0.938	35.29	0.489
Ctry=JP	5.71	**40**	**0.024****	**0**	**0.051***	8.33	0.671	5.88	0.927
Ctry=USA	51.43	20	0.184	52.78	0.821	50	0.917	58.82	0.501
Exp.Typ=neur.gen	48.57	60	0.633	50	0.811	41.67	0.619	47.06	0.892
Exp.Typ=neur.NM.Exp	51.43	40	0.633	50	0.811	58.33	0.619	52.94	0.892
NMD.Ctr=NMD.Ctr_Yes	70	**20**	**0.028****	69.44	0.921	75	0.714	82.35	0.217
Pt.mo=+20pt	44.29	40	0.868	38.89	0.363	33.33	0.427	**64.71**	**0.061***
Pt.mo=<5pt	7.14	20	0.355	8.33	0.724	8.33	0.822	0	0.237
Pt.mo=5-20pt	48.57	40	0.724	52.78	0.482	58.33	0.48	35.29	0.225
Tim.Exp=+10yr	70	60	0.63	63.89	0.267	75	0.714	82.35	0.217
Tim.Exp=<5yr	7.14	20	0.355	8.33	0.724	0	0.379	5.88	0.886
Tim.Exp=5-10yr	22.86	20	0.941	27.78	0.332	25	0.828	11.76	0.229
Wk.pl.nt4p=Wk.pl.nt4p_Yes	2.86	0	0.861	0	0.232	8.33	0.343	5.88	0.486
Wk.pl.nUni=Wk.pl.nUni_Yes	11.43	0	0.535	16.67	0.182	16.67	0.547	**0**	**0.094***
Wk.pl.Priv=Wk.pl.Priv_Yes	28.57	60	0.158	22.22	0.242	25	0.799	35.29	0.494
Wk.pl.public=Wk.pl.public_Yes	14.29	20	0.695	11.11	0.461	8.33	0.585	23.53	0.248
Wk.pl.Uni=Wk.pl.Uni_Yes	58.57	20	0.1	63.89	0.367	58.33	0.978	58.82	0.988
Wk.set.Amb=Wk.set.Amb_Yes	75.71	100	0.237	69.44	0.225	58.33	0.155	**94.12**	**0.04****
Wk.set.DH=Wk.set.DH_Yes	24.29	0	0.237	22.22	0.69	33.33	0.443	29.41	0.579
Wk.set.HoWd=Wk.set.HoWd_Yes	64.29	80	0.51	55.56	0.127	75	0.425	70.59	0.557
Wk.set.LogoCtr=Wk.set.LogoCtr_Yes	5.71	20	0.285	**0**	**0.051***	0	0.463	**17.65**	**0.044****
Wk.set.OccCtr=Wk.set.OccCtr_Yes	4.29	20	0.214	0	0.109	0	0.564	11.76	0.157
Wk.set.Oth=Wk.set.Oth_Yes	1.43	0	0.929	2.78	0.514	0	0.829	0	0.757
Wk.set.Phy.Reh=Wk.set.Phy.Reh_Yes	10	20	0.494	**0**	**0.004****	16.67	0.436	**23.53**	**0.062***

Table S4 - Extracted "Characteristic" questions with the recoded labels

Code	Domain/Question	Possible answers	HCPC assigned code
Q1	Provenience	USA	Ctry=USA
		EU	Ctry=EU
		JP	Ctry=JP
Q2	Expert typology	General Neurologist	Exp.Typ=neur.gen
		Neuromuscular disease expert	Exp.Typ=neur.NM.Exp
Q3	Do you work in a NMD center?	Yes / No	NMD.Ctr=Yes/No
Q4	Select the typology of your workplace (more than one allowed)	University center	Wk.pl.Uni=Yes/No
		Not University center	Wk.pl.nUni=Yes/No
		Private center	Wk.pl.Priv=Yes/No
		Private not for profit	Wk.pl.nt4p=Yes/No
		Public	Wk.pl.public=Yes/No
Q5	Select the context in which you work (more than one allowed)	Ambulatory	Wk.set.Amb=Yes/No
		Day hospital	Wk.set.DH=Yes/No
		Hospital ward	Wk.set.HoWd=Yes/No
		Occupational therapy center	Wk.set.OccCtr=Yes/No
		Physiotherapy /rehabilitation center	Wk.set.Phy.Reh=Yes/No
		Logopedic center	Wk.set.LogoCtr=Yes/No
Q6	Select the year/experience in treating MG patients	Less than 5 years	Tim.Exp.=5.yr
		Between 5 and 10 years	Tim.Exp.=5-10yr
		More than 10 years	Tim.Exp.=+10yr
Q7	Select the mean number of patients that you follow on a regular monthly basis	Less than 5 patients	Pt.mo=<5pt
		Between 5 and 20 patients	Pt.mo=5-20pt
		More than 20 patients	Pt.mo=+20pt

Table S5 - Extracted "Opinion" questions with the recoded labels (N.Ap = not appropriateness; BN = Bottlenecks; Set.= Setting; Cpl. = Completeness)

Sub Process	Based on your experience, do you find any **not appropriate** activity in the subprocess?	Based on your experience, do you think the process flow is **complete**?	Based on your practical experience could you find the most relevant **bottleneck**?	What is the most **common setting** in which the process is currently executed?
Diagnosis (1)	Q8<br>N.Ap.1=Yes/No	Q11<br>Cpl.1=Yes/No	Q13<br>BN.1=Yes/No	Q16<br>Set.1=Mono/Multi
Pharmacologic therapy (2)	Q18<br>N.Ap.2=Yes/No	Q21<br>Cpl.2=Yes/No	Q23<br>BN.2=Yes/No	Q26<br>Set.2= Mono/Multi
Myasthenic crisis (3)	Q28<br>N.Ap.3=Yes/No	Q31<br>Cpl.3=Yes/No	Q33<br>BN.3=Yes/No	Q36<br>Set.3= Mono/Multi
Speech/Swallow (4)	Q38<br>N.Ap.4=Yes/No	Q41<br>Cpl.4=Yes/No	Q43<br>BN.4=Yes/No	Q46<br>Set.4= Mono/Multi
Occupational/Physiotherapy (5)	Q48<br>N.Ap.5=Yes/No	Q51<br>Cpl.5=Yes/No	Q53<br>BN.5=Yes/No	Q56<br>Set.5= Mono/Multi
Psychological (6)	Q58<br>N.Ap.6=Yes/No	Q61<br>Cpl.6=Yes/No	Q63<br>BN.6=Yes/No	Q66<br>Set.6= Mono/Multi
Lifestyle (7)	Q68<br>N.Ap.7=Yes/No	Q71<br>Cpl.7=Yes/No	Q73<br>BN.7=Yes/No	Q76<br>Set.7= Mono/Multi

Figure S6 - Heatmap of the characteristic variables of the clusters of experts

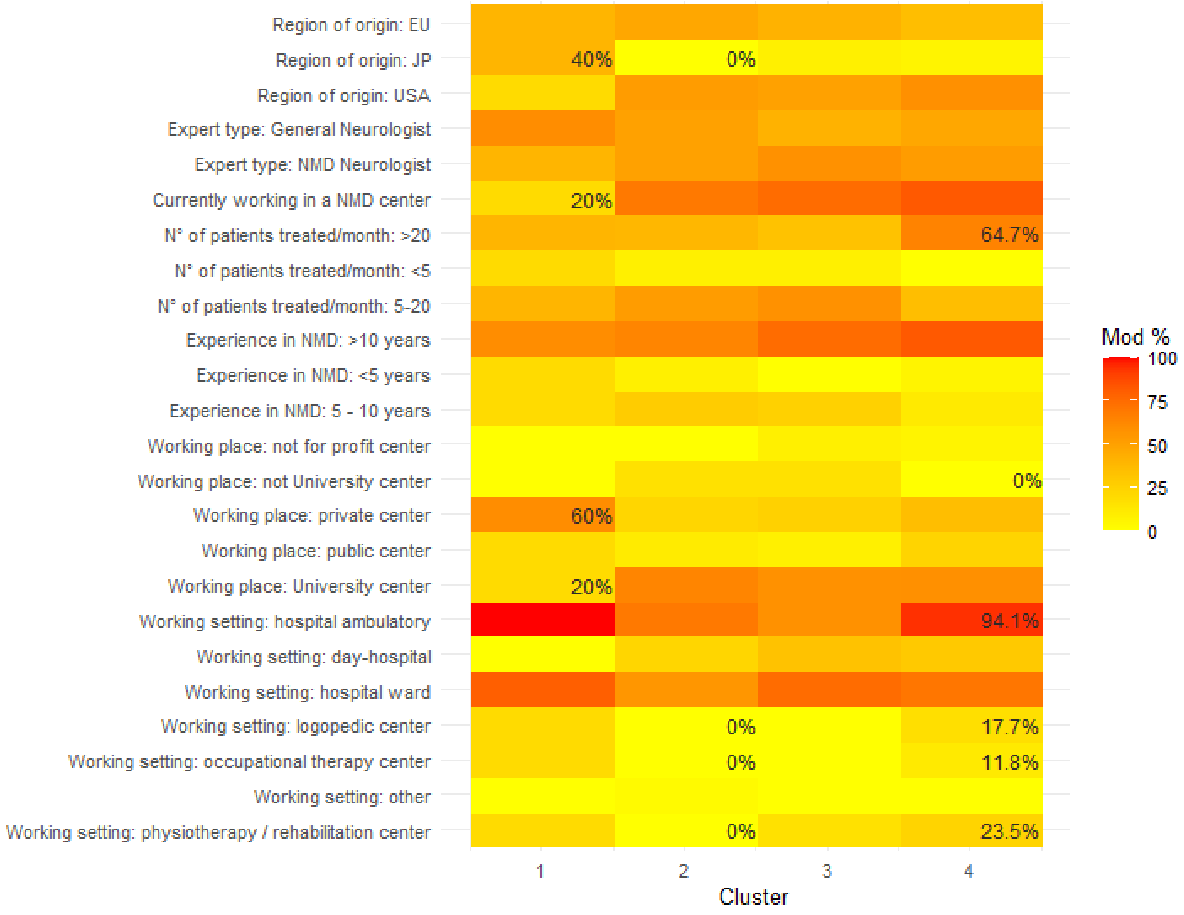

Supplement: Supplementary file 2 — Additional file 2: Table S1. Contribution of the “opinion” variables to the dimensions with the rank of the first ten variables. Table S2. Squared cosine (cos2 ) of the "opinion" variables. Figure S1. Scree plot of the results of MCA. Figure S2. Contribution of variables to dimension 1. Figure S3. Contribution of variables to dimension 2. Figure S4. Contribution of variables to dimension 3. Figure S5. HCPC resulting dendrogram. Table S3. Original output table from factomineR HCPC results with the assigned codes. Table S4. Extracted "Characteristic" questions with the recoded labels. Table S5. Extracted "Opinion" questions with the recoded labels (N.Ap = not appropriateness; BN = Bottlenecks; Set.= Setting; Cpl. = Completeness). Figure S6. Heatmap of the characteristic variables of the clusters of experts. [file 12913_2023_9139_MOESM2_ESM.pdf]
